# Computational Metabolomics Tools Reveal Metabolic Reconfigurations Underlying the Effects of Biostimulant Seaweed Extracts on Maize Plants under Drought Stress Conditions

**DOI:** 10.3390/metabo12060487

**Published:** 2022-05-27

**Authors:** Morena M. Tinte, Keabetswe Masike, Paul A. Steenkamp, Johan Huyser, Justin J. J. van der Hooft, Fidele Tugizimana

**Affiliations:** 1 Department of Biochemistry, University of Johannesburg, Auckland Park, Johannesburg 2006, South Africa; morenatinte@gmail.com (M.M.T.); keabetswemasike@yahoo.com (K.M.); psteenkamp@uj.ac.za (P.A.S.); 2 Omnia Group, International Research and Development Division, Ltd., Bryanston, Johannesburg 2021, South Africa; johan.huyser@omnia.co.za; 3 Bioinformatics Group, Wageningen University, 6708 PB Wageningen, The Netherlands

**Keywords:** biostimulants, seaweed extracts, metabolomics, molecular networking, GNPS, MS2LDA substructure discovery, pathway analysis, maize, drought, abiotic stress

## Abstract

Drought is one of the major abiotic stresses causing severe damage and losses in economically important crops worldwide. Drought decreases the plant water status, leading to a disruptive metabolic reprogramming that negatively affects plant growth and yield. Seaweed extract-based biostimulants show potential as a sustainable strategy for improved crop health and stress resilience. However, cellular, biochemical, and molecular mechanisms governing the agronomically observed benefits of the seaweed extracts on plants are still poorly understood. In this study, a liquid chromatography–mass spectrometry-based untargeted metabolomics approach combined with computational metabolomics strategies was applied to unravel the molecular ‘stamps’ that define the effects of seaweed extracts on greenhouse-grown maize (*Zea mays*) under drought conditions. We applied mass spectral networking, substructure discovery, chemometrics, and metabolic pathway analyses to mine and interpret the generated mass spectral data. The results showed that the application of seaweed extracts induced alterations in the different pathways of primary and secondary metabolism, such as phenylpropanoid, flavonoid biosynthesis, fatty acid metabolism, and amino acids pathways. These metabolic changes involved increasing levels of phenylalanine, tryptophan, coumaroylquinic acid, and linolenic acid metabolites. These metabolic alterations are known to define some of the various biochemical and physiological events that lead to enhanced drought resistance traits. The latter include root growth, alleviation of oxidative stress, improved water, and nutrient uptake. Moreover, this study demonstrates the use of molecular networking in annotating maize metabolome. Furthermore, the results reveal that seaweed extract-based biostimulants induced a remodeling of maize metabolism, subsequently readjusting the plant towards stress alleviation, for example, by increasing the plant height and diameter through foliar application. Such insights add to ongoing efforts in elucidating the modes of action of biostimulants, such as seaweed extracts. Altogether, our study contributes to the fundamental scientific knowledge that is necessary for the development of a biostimulants industry aiming for a sustainable food security.

## 1. Introduction

Recurring drought conditions are projected to reduce the global production of major crops by approximately 50% by 2050, and 90% by 2100 [1,2]. The global production of maize (*Zea mays* L.) has declined by 40% due to drought in the past few decades and is expected to further decrease by 10–25% with a 1 °C increase in global surface temperatures [2,3]. Maize is a cereal crop species of global economic significance that contributes to approximately 12% of the world’s food demand and ranks first in grain global production at a volume of 1.135 billion tons. Recurring droughts, therefore, pose a significant threat to global food security and economies [4,5,6]. Over the past few years, crop protection and productivity was achieved by the extensive application of synthetic chemical fertilizers and pesticides. However, these agrochemicals have both short- and long-term detrimental effects on the natural ecosystem, as well as on human health [7,8,9]. Hence, eco-friendly, sustainable, and innovative strategies that are capable of improving crop productivity and drought tolerance, without compromising soil and human health, are imperatively needed [6,10].

Biostimulants have emerged as promising eco-friendly alternatives to traditional agrochemicals, with evidence of enhancing crop productivity and tolerance to abiotic stressors, such as drought [9,10]. Biostimulants are defined as biological formulations that improve plant health and productivity through the stimulating action of novel and/or emergent properties within the complex natural mixture [8,10]. The common basis of biostimulant formulations are plant growth promoting rhizobacteria (PGPR), humic and fulvic acids, protein hydrolysates, chitosan and biopolymers, and seaweed extracts [6,11]. Seaweed extracts are rich sources of bioactive phenolic compounds, polysaccharides, phytohormones, amino acids, and macro- and micro-element nutrients that are capable of inducing changes in the physiological and biochemical processes involved in plant nutrient uptake and growth [7,12]. For example, *Kappaphycus alvarezii* seaweed extract upregulated genes involved in enhancing root growth, auxin signaling, nitrogen metabolism, and antioxidant activity, thus improving the root growth, grain yield, and nutrient content of maize roots to mitigate drought stress [5]. Seaweed-based biostimulants account for more than 33% of the global biostimulant market and are projected to reach a value of EUR 894 million by 2022 [12]. The above-mentioned biostimulants were proposed to induce the metabolic reprogramming of plants. Up to now, however, the molecular mechanisms through which biostimulants act, and which maize metabolic changes and pathways are involved in response to the biostimulants’ presence, have remained unknown [10,13].

To investigate the metabolic perturbations caused by biostimulants, herein we report the application of a liquid chromatography–tandem mass spectrometry (LC–MS/MS)-based untargeted metabolomics approach to unravel the mechanistic effects of a seaweed-based biostimulant on the metabolism of maize plants under normal and drought conditions. Molecular networking (MN) tools, in the Global Natural Products Social Molecular Networking (GNPS) ecosystem [14,15] were applied to process and analyze the generated spectral data. These MN strategies enable a broad overview of the molecular information that can be inferred from the MS/MS data. This analysis of chemical relationships between every MS/MS spectrum, visualizing the entire metabolome detected in a sample using the MolNetEnhancer approach, reveals structurally related molecular families in maize plants [16,17,18,19]. Furthermore, the implementation of the Metabolomics Pathway Analysis (MetPA) tool, a MetaboAnalyst feature, aids in identifying the most relevant pathways within the metabolome [20,21]. Thus, profiling the metabolome of maize plants, treated with a seaweed formulation, would reveal the metabolic reprogramming due to seaweed treatment. Such insights would provide a fundamental understanding of the molecular mechanisms induced by seaweed-based biostimulants towards plant growth promotion and enhanced defenses. Furthermore, it would help us to create a roadmap for novel biostimulant formulation and strategies for sustainable agricultural practices.

## 2. Results and Discussion

Seaweed extracts, the same as other plant biostimulants, are currently considered novel and sustainable strategies in the agro-industry. Seaweed extracts were shown to mitigate abiotic stress and enhance plant productivity [5]. However, the effects of biostimulants on the plant metabolism and overall underlying molecular mechanisms that govern the positive effects of biostimulants, such as improved nutrient uptake, increased ROS scavenging and plant height, and tolerance to abiotic stress, remain enigmatic [11,22,23]. In this study, we applied an LC–MS/MS-based untargeted metabolomics study to unravel the effects of the seaweed-based biostimulant (with soil- and foliar application) on the metabolism of maize plants under normal and drought conditions.

**Figure 1:**
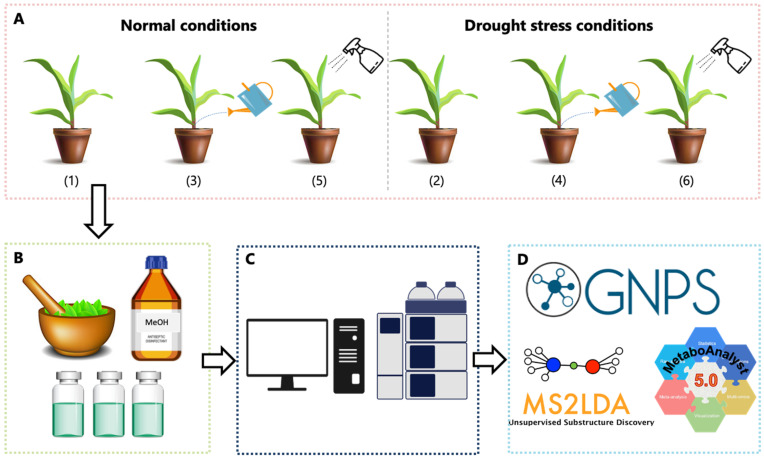
Schematic overview of the study design. (**A**) Maize plants were divided into 6 groups, with each group comprised of 5 maize plants (biological replicates) to make up a total of 30 plant samples. Control groups consisted of untreated maize plants under (1) normal and (2) drought stress conditions. Treatment groups consisted of soil application of seaweed-based biostimulant under (3) normal and (4) drought stress conditions, and foliar application of seaweed-based biostimulant under (5) normal and (6) drought stress conditions. Leaves were harvested at four time points (1 day, 3-, 7-, and 14 days after treatment) followed by (**B**) metabolite extraction and sample preparation; (**C**) Extracts were analyzed with liquid-chromatography mass-spectrometry (LC–MS/MS); (**D**) Data analysis of the acquired LC-MS data was mined using chemometrics and computational tools, such as GNPS, MS2LDA and MetaboAnalyst.

A schematic overview of the study design is illustrated in Figure 1. The study was designed to comprise maize leave extracts from control and treatment plant groups. The control group consisted of untreated maize plants under (1) normal (water level maintained at 90% plant available water [PAW]) and (2) drought stress (water level maintained at 50% PAW) conditions, whereas the treated group was soil-applied biostimulant-treated maize plants under (3) normal and (4) drought stress conditions, foliar-applied biostimulant-treated maize plants under (5) normal and (6) drought stress conditions. In the final metabolomics dataset, each group consisted of five biological replicates and three technical replicates. The data acquired from the untargeted LC–MS/MS analyses of methanol extracts from maize leaves was mined and interpreted using various chemometrics and computational approaches, such as feature-based molecular networking (FBMN), MS2LDA, Network Annotation Propagation (NAP) in silico annotation tool, and MolNetEnhancer. The MetaboAnalyst generated an unsupervised principal component analysis (PCA) plot that provided a global visualization of the data and revealed sample groupings related to drought stress conditions, biostimulant treatment, and its method of application (soil vs. foliar application) (Figure S1).

### 2.1. A Molecular Networking Approach for the Annotation and Visualization of the Extracted Maize Metabolome

Molecular networking (MN) organizes metabolites or experimental spectra into molecular families/classes, based on spectral similarities, followed by metabolite annotation and identification [15,24]. Thereby, molecular families/classes with unknown metabolites can be distinguished when connected to similar or related annotated metabolites. Feature-based MN (FBMN), unlike ‘classical MN’, incorporates MS1 chromatographic and quantitative information, such as retention time, isotope patterns, ion mobility separation, and peak heights or areas, thereby enabling the accurate detection of isomers producing similar MS2 in molecular networks and the use of downstream statistical analysis and annotation tools [15,25].

**Figure 2:**
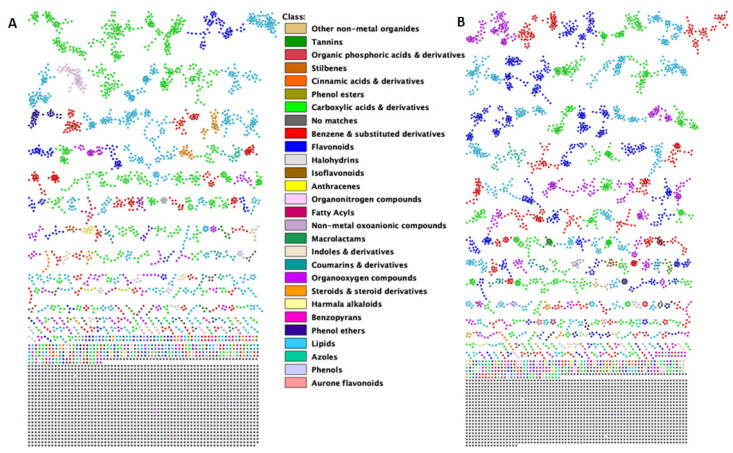
MolNetEnhancer enriched feature-based molecular networks of (**A**) positive electrospray ionization (ESI+) and (**B**) negative electrospray ionization (ESI−) MS/MS spectra obtained from maize leave extracts. The MolNetEnhancer enriched molecular networks depict structurally similar nodes as molecular families/clusters. GNPS spectral library matched annotations and in silico Network Annotation Propagation (NAP) annotations were used to predict chemical compound class annotations, represented by colored nodes (ClassyFire class ontology terms) with nodes without class annotation colored grey.

FBMN was employed through the GNPS infrastructure to explore and visualize the acquired MS/MS spectra data from the maize leave extracts. The FBMN job links are attached in the Supplementary Materials. The molecular network generated from the positive dataset consists of 4648 nodes of which 68 nodes (green nodes, Figure S2A, Supplementary Materials) were annotated or matched with metabolites within the GNPS library mass spectral databases (Table S1, Supplementary Materials). The molecular network for the negative dataset comprised of 4910 nodes of which 61 nodes (the green nodes in Figure S2B, Supplementary Materials) were annotated or matched with the metabolites within the GNPS library mass spectral databases (Table S1, Supplementary Materials). The GNPS library databases consist of mass spectra acquired from the use of various sample preparation protocols and mass spectrometers, hence, the variation in the mass spectra quality and content [24]. Moreover, some of these mass spectra lack chemical standards and therefore limit the accurate annotation of the metabolites [24]. Hence, manual inspection is sometimes required to confirm the putative (semi-automated) annotations and predictions [24]. In addition to the FBMN metabolite annotations, 33 metabolites such glucogallin, kaempferol-7-O-hexoside, phenylalanine, and tryptophan were manually annotated (Table S2), as described in the experimental Section 3.6.1. FBMN was used in combination with the MolNetEnhancer workflow to allow for an efficient exploration of the structural diversity and distribution of the different chemical classes in the extracted maize metabolome (Figure 2). The outputs from FBMN and MolNetEnhancer revealed that the measured maize metabolome was comprised of various metabolite classes, including carboxylic acid, flavonoid, benzene, lipid, organic acid, cinnamic acid, and amino acid metabolites (Figure 2 and Tables S1 and S2, Supplementary Materials).

The carboxylic acids and derivatives, and the lipid metabolite classes were widely observed in the positive ionization mode data (Figure 2A), in contrast to the negative ionization mode data, which were observed to be rich in flavonoids, benzene and substituted derivatives, and organooxygen compounds (Figure 2B). Zooming in, the carboxylic acids and derivatives’ metabolite class consists of amino acids, such as the annotated isoleucine, phenylalanine, and tryptophan metabolites (Figure S3 and Tables S1 and S2, Supplementary Materials). These metabolites were reported to increase under drought stress, with phenylalanine and tryptophan reported to be widely accumulated in maize leaves [26]. Various terpenoid and steroid glycoside-related metabolites were observed within the lipid class. Additionally, the benzene and substituted derivatives class was observed to comprise of halobenzenes and hydroxycoumarin metabolites, whereas the flavonoid class consisted largely of flavonoid glycosides and organooxygen compounds, such as DIBOA-glucoside, apigenin-7-O-glucoside, and rutin subclasses (Figure S3 and Tables S1 and S2, Supplementary Materials). DIBOA-glucoside was reported to increase under stress conditions [27]. Finally, rutin and apigenin-7-O-glucoside are considered to be great antioxidants that decrease oxidative damage and enhance tolerance to stresses, such as drought [28,29].

**Figure 3:**
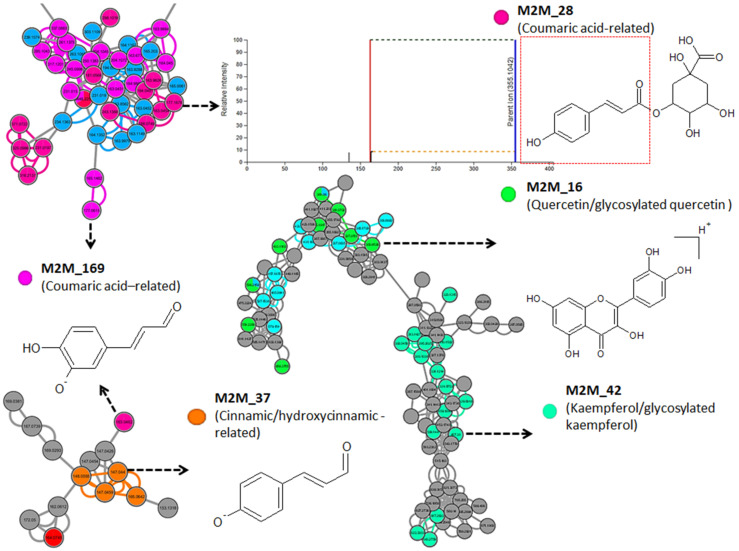
Characterized Mass2Motifs mapped on the spectral molecular network of the positive electrospray ionization (ESI+) MS/MS spectra obtained from maize leave extracts. Colored nodes represent the distinct Mass2Motif annotations and their occurrence within the spectra dataset. The shared Mass2Motifs among the various nodes were mapped on the edges connecting the nodes.

To explore the substructural diversities of the metabolites within the datasets, MS2LDA was applied to explore, aiding in the metabolite annotation and/or functional classification of unannotated metabolites within the extracted maize metabolome (Figure 2). Previously annotated Mass2Motifs, which are common patterns of mass fragments and neutral losses from MotifDB, were used in the exploration of the substructural diversity and identification of known chemistry and recognition of yet unknown chemistry. Five hundred and forty-one (541) Mass2Motifs were discovered from the positive, and 181 from the negative datasets, of which 134 and 27, respectively, were annotated Mass2Motifs derived from the GNPS, MassBank, Euphorbia, Rhamnaceae Plant, Streptomyces and Salinisporus, Photorhabdus, and Xenorhabdus MotifSets included in MotifDB. Fifty-four (54) and 18 of the previously annotated Mass2Motifs in the positive and negative ionization mode datasets, respectively, had a degree of five or higher. Piperazine, ferulic, cinnamic/hydroxycinnamic, kaempferol/glycosylated kaempferol, flavonoid, coumaric, and rhamnocitrin-related substructures are examples of the Mass2Motifs, with assigned fragmentation spectra, extracted from the positive dataset (Figure 3). The Mass2Motifs discovered in the maize metabolomics data are indicative of a diverse and complex chemistry of the maize metabolome, spanning a range of biological pathways and chemical compound classes. The exploration of the fragmentation spectra by MS2LDA, hence, provided metabolome-wide insights into the specialized maize metabolism through the discovery of Mass2Motifs that are related to plant metabolite structures, as depicted in Figure 3. Furthermore, these substructure annotations support putative *de novo* structural metabolite annotations, thus accelerating the annotation of metabolites and providing meaningful biochemical interpretation that aids in the understanding of plant mechanisms. However, to fully capture the maize metabolic diversity and enhance our understanding of the plant’s mechanisms, the unknown motifs should be explored and annotated in the future. MS2LDA is thus limited by the vast amount of unannotated Mass2Motifs, that require expert knowledge for structural annotation, which makes this process laborious [17,19].

**Figure 4:**
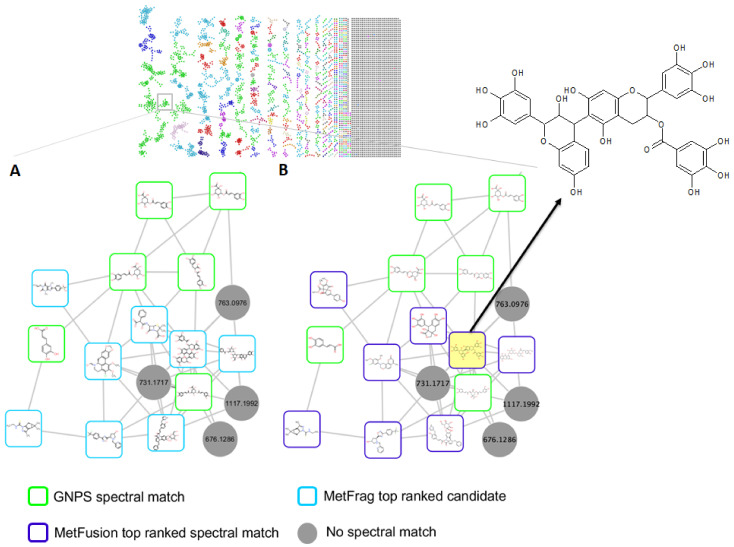
Molecular network family cluster of the carboxylic acid and derivatives chemical compound class containing GNPS spectral library and NAP propagated annotations from maize leave extracts analyzed in positive electrospray ionization (ESI+). (**A**) Cluster of polyphenolic flavanol compounds matched with GNPS spectral library (green square-shaped nodes), top ranked predicted NAP MetFrag candidate structures (blue square-shaped nodes) and no spectral match (grey nodes) annotations; (**B**) Cluster of polyphenolic flavanol compounds matched with GNPS spectral library (green square-shaped nodes), NAP MetFusion top ranked candidate structures, with epigallocatechin-(4β->8)-epigallocatechin-3-O-gallate (*m*/*z* 763.1514) compound highlighted in yellow, (purple square-shaped nodes) and no spectral match (grey nodes) annotations.

**Figure 5:**
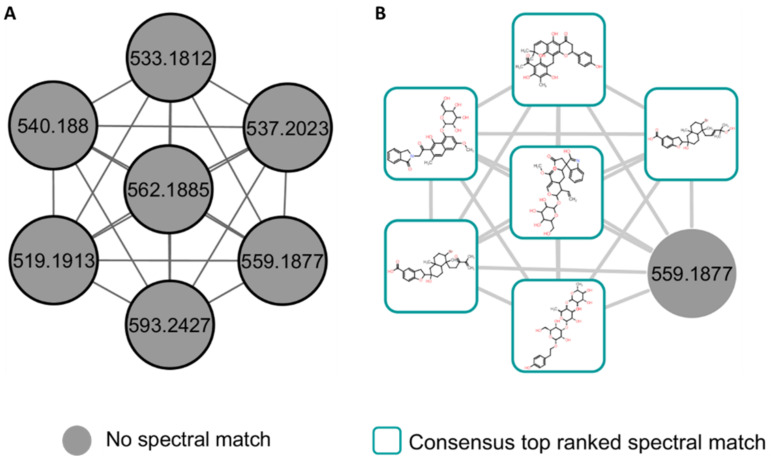
Molecular network family clusters of unidentified chemical compound class from maize leave extracts analyzed in positive electrospray ionization (ESI+). (**A**) Molecular network family with unknown structural annotations (grey nodes) and the resultant (**B**) top ranked NAP Consensus structural annotations (cyan nodes).

**Figure 6:**
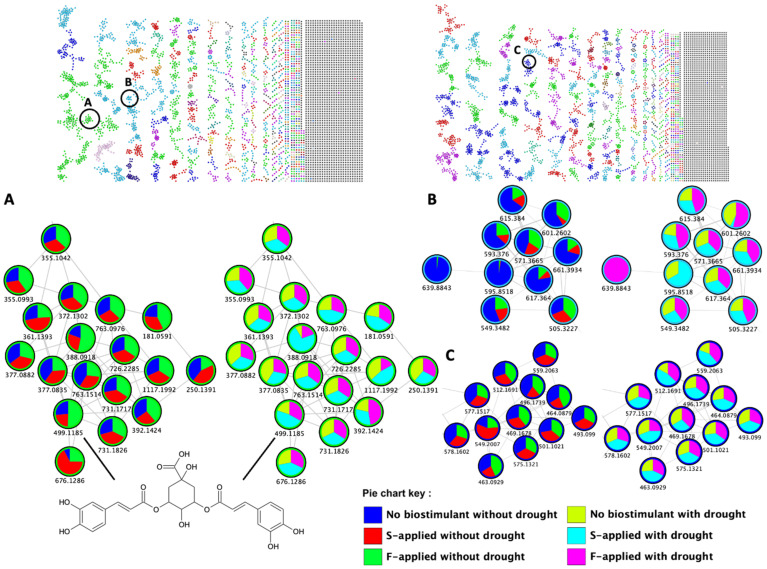
The MolNetEnhancer molecular network of positive electrospray ionization (ESI+) (top left) and negative ESI- MS/MS (top right) spectra obtained from maize leave extracts. The enriched molecular networks depict structurally similar nodes as molecular families/clusters, with the annotated metabolites, MS2LDA substructures and NAP annotations assigned class annotations, represented by colored nodes and nodes with no class annotation as grey. The pie charts show the differential effects of the seaweed-based biostimulant treatment and their method of application on the metabolite levels of the (**A**) carboxylic acid and derivatives; (**B**) lipids; and (**C**) flavonoid molecular families under normal and drought stress conditions. Abbreviations: S-applied, soil applied; and F-applied, foliar applied.

Furthermore, to support the MolNetEnhancer chemical compound class annotation (Figure 2), the Network Annotation Propagation (NAP) in silico annotation tool was applied (Figure 4 and Figure 5). NAP predicts and re-ranks candidate structure annotations from the compound databases, based on the predicted similarity of the experimental MS/MS data [15,17]. In the case where there is a node with a spectral library match within a molecular family, NAP utilizes the Fusion scoring method to predict and re-rank the candidate structures. The Fusion scoring method utilizes the MetFrag tool to produce in silico fragmentation predictions, which are subsequently combined with the output of the spectral library search by the MetFusion tool to improve the candidate structure ranking for direct neighbor nodes based on the structural similarity of library matches and in silico candidate structures. The structural annotations of these nodes within the molecular network are represented by the top MetFusion spectral library matches [30]. For instance, in the highlighted section of the carboxylic acids and the derivatives molecular family cluster (Figure 4), eight nodes were propagated from known GNPS structural annotations, using the NAP Fusion scoring method. The top ranked MetFrag and MetFusion candidate structures from the GNPS, Dictionary of Natural Products (DNP), Super Natural II (SUPNAT), Chemical Entities of Biological Interest (ChEBI), and MarinLit databases are, respectively, depicted in Figure 4A,B. Among the top MetFusion candidate structures depicted in Figure 4B (yellow highlighted structure/node) is epigallocatechin-(4β->8)-epigallocatechin-3-O-gallate (*m*/*z* 763.1514), an esterified epigallocatechin-3-O-gallate polyphenolic flavanol/catechin, which was accumulated in plant species under drought conditions, due to its high antioxidant activity [31,32]. This metabolite was observed to have increased in drought-stressed plants treated with the seaweed-based biostimulant (Figure 6A).

Moreover, when there are no spectral library matches within a molecular family, as depicted on the observed molecular family cluster in Figure 5A, NAP utilizes network consensus scoring to re-rank the MetFrag predicted in silico candidate structures, based on the structural similarity between the connected nodes, thereby propagating candidate structures for the connected nodes [30,33]. NAP with consensus scoring for the illustrated molecular family in Figure 5A, resulted in the propagation of six out of seven nodes, including the polyphenolic compounds ligurobustoside M and 4′-hydroxyisorottlerin among the top ranked candidate structures (Figure 5B). NAP thus accelerates and improves the structural annotations of unknown metabolites by propagating the structural information of any node(s) within a molecular family/cluster to other or neighboring nodes within that family/cluster, [25]. NAP is, however, limited by the possible inaccuracy of the MetFrag tool in predicting complex rearrangement reactions, due to its bond disconnection approach in generating fragment ions [34,35]. Furthermore, NAP is limited to small metabolites [18,34]. Thus, the expert inspection of the NAP predicted candidates is encouraged to ensure accurate structural annotation [30]. The NAP-acquired structural annotations also provide input for the chemical compound class information of the nodes in the molecular network that is used by the MolNetEnhancer workflow, and that thereby illuminates the maize metabolome, thus enhancing insight into the maize metabolome and aiding in biochemical interpretation.

Thus, in our study, we applied a combination of the FBMN, unsupervised MS2LDA substructure discovery, NAP in silico annotation tool, and the ClassyFire automated chemical classification tool outputs, coming together in the MolNetEnhancer workflow (Figure 2). ClassyFire utilizes structural features for the automated prediction of a metabolite’s chemical classification, therefore chemical class annotation through ClassyFire is limited by the drawbacks of spectral matching and in silico annotation tools [17,36]. The benefit of using ClassyFire within the MolNetEnhancer, is that it allows for automated chemical class annotations based on library matches and candidate structures, and a hierarchical ontology. Of course, this will not always lead to “correct” chemical class annotations, as the ClassyFire ontology is sometimes different from a field-specific ontology (Tables S1 and S2, Supplementary Materials). Thus, MolNetEnhancer allowed a comprehensive exploration and enrichment of chemical annotations, discovering the subtle substructural diversity within molecular families. Furthermore, assessing the features that chemometrically differentiate treatment groups (Table S3, Supplementary Materials), the molecular networking approach allowed the annotation of more than 50% of these features (Figure S4, Supplementary Materials).

**Figure 7:**
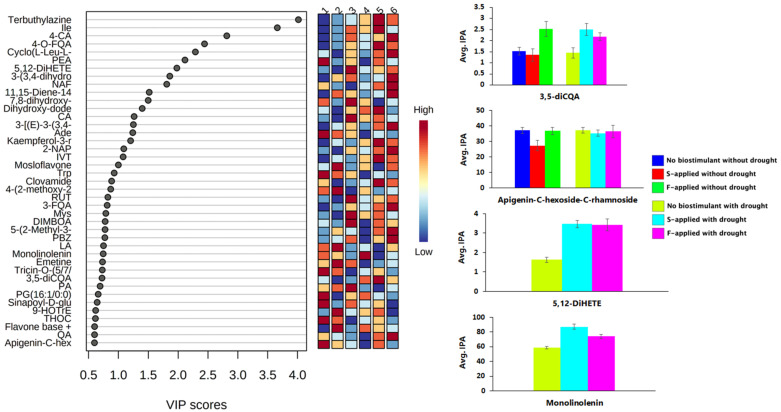
Visualization of discriminatory metabolites and concentration of selected metabolites in individual groups. PLS-DA VIP score plot of important identified metabolites with their concentration in each group displayed by the heatmap, and bar graphs displaying the average peak intensities of selected metabolites in each group. Abbreviation: S-applied, soil applied; F-applied, foliar applied; and Avg. IPA, averaged integrated peak area. Refer to Tables S1 and S2, Supplementary Materials, for full names of metabolites.

Thus, this MN strategy allowed the visualization of molecular families with class annotations [36] (Figure 2, Tables S1 and S2) and quantitative descriptions of the individual metabolites (Figure 6). For instance, the maize metabolome was observed to broadly consist of the carboxylic acid and derivatives, flavonoid, benzene and substituted derivatives, and lipid molecular families (Figure 2). The quantitative data are depicted by pie charts that reflect the differential effects of the seaweed-based biostimulant treatments and the method of application on individual metabolites under both normal and drought stress conditions (Figure 6). For example, in the carboxylic acid and derivatives molecular family cluster, a quantitative description (i.e., pie chart) of 3,5-dicaffeoylquinic acid (*m*/*z* 499.1185, Figure 6A) highlighted the effect of the seaweed-based biostimulant treatments on its distribution, with the greatest effect under normal conditions observed by the foliar application method. In contrast, under drought stress conditions, the soil application method was shown to have the greatest effect on the distribution of 3,5-dicaffeoylquinic acid in comparison to the foliar-applied biostimulant. However, in the depicted lipid molecular family, the foliar application method was observed to have the greatest effect on the distribution of the individual metabolites, in comparison to the soil application method, under both normal and drought stress conditions (Figure 6B). A similar effect was observed for some of the individual metabolites within the flavonoid molecular family for plants under normal conditions, whereas in drought stress conditions, the soil application method was observed to have the greatest effect on the distribution of the individual metabolites (Figure 6C and Figure 7). Below, several chemical compound classes observed in the maize metabolomics data are described in more detail.

The flavonoid molecular class is regarded as one of the largest class of polyphenolic metabolites in plant species, as is also reflected in Figure 2 (in particular in the negative ionization mode network), which encompasses approximately 8000 metabolites contributing to plant processes involved in the development, plant–environment interactions, and defenses [10,29,37]. In maize, various flavonoids were reported to be synthesized and widely distributed in leaf bases and tips, in response to abiotic stresses such as drought [38,39]. Flavonoids can function as antioxidants that reduce and protect cells from oxidative damage caused by abiotic stress-induced reactive oxygen species (ROS) [29,40]. In our MolNetEnhancer networks, we observe a broad detection of flavonoid metabolite features that vary in distribution with regards to the environmental conditions they are linked to, and the possible role of the individual flavonoid metabolite in the maize plant’s processes. For example, as previously stated by [28], apigenin is one of the best ROS scavengers in drought conditions, and we observe an increase in apigenin-C-hexoside-C-rhamnoside (*m*/*z* 577.1517) and hyperoside (*m*/*z* 463.0929) metabolites in drought-stressed maize plants treated with the seaweed-based biostimulant (Figure 6C and Figure 7 and Tables S1 and S2). The flavonoid pathways are key branches of the phenylpropanoid metabolism and can thus direct the metabolic flux towards the synthesis of other specialized metabolic pathways and molecular families, such as organic compounds [29].

Organic compounds are utilized for plant growth and tolerance to stress, by promoting nutrient (i.e., mineral) uptake, stabilizing the activity of the tricarboxylic acid (TCA) cycle, and maintaining pH and redox balance in cells. Thereby, organic compounds aid in the reduction of stress-induced oxidative stress [41,42]. Additionally, the benzenoid organic compounds function as chemical signals and precursors for natural products involved in plant fitness [43,44,45]. The biosynthesis, distribution, up- and downregulation of organic compounds are based on the plants’ response to the stress condition [42]. This may therefore explain their broad existence within the extracted maize metabolome (Figure 2). The organic compound molecular families (i.e., carboxylic acid and derivatives, benzene and substituted derivatives, and organooxygen compounds) (Figure 2) are thus essentially important to plants as they function in various cellular metabolic processes [42].

Lipids have diverse functions in plants that include their influence on the performance, regulation, and physical properties of plant membranes. Furthermore, lipids function as integral components of the photosynthetic protein complexes of the electron transport chain and serve as signaling molecules that regulate cell metabolism [46,47]. The observed lipid molecular families (Figure 2) were associated with the maintenance of cell membrane stability, energy metabolism, and antioxidant activity [48,49]. Furthermore, lipids were reported to have significantly increased in the drought-stressed plants, thus suggestive of their role in plant abiotic stress responses [50]. This may, therefore, explain the observed increase in fatty acyl lipids, including 5,12-DiHETE and monolinolenin, in drought-stressed plants treated with the seaweed-based biostimulant (Figure 6B and Figure 7).

The partial least squares discriminant analysis’ (PLS-DA) variable importance in projection (VIP) provided visualization of the top annotated metabolites (i.e., the metabolites with a VIP > 1.0) that contributed to the variation of the sample groups (Figure 7, Tables S1 and S2, Supplementary Materials). A total of 20 metabolites were discovered as significant metabolites that contributed to the group variations. The metabolites included stress defense-related metabolites, such as isoleucine, coumaric acid, caffeic acid, adenine, kaempferol-3-rutinoside, and isovitexin (Figure 7). The roles that some of these metabolites play in plant productivity and/or stress tolerances, are discussed in Section 2.2. Moreover, their concentration and other individual metabolites for each group are illustrated in the heatmap (Figure 7).

The quantitative analyses of individual metabolites, visualized within the molecular networks and heatmap (Figure 7), showed the effect of the seaweed-based biostimulant treatment on metabolite distribution. The majority of the molecular family metabolites were increased in abundance by the seaweed-based biostimulant treatment, with the foliar application having the greatest effect on the increased distribution of most metabolites under normal conditions (Figure 6 and Figure 7). On the contrary, under drought stress conditions, the soil application method was observed to be superior to the foliar application method in increasing the distribution of most metabolites, in particular those that are associated with stress tolerance (Figure 6 and Figure 7). Thus, it can be suggested that the seaweed-based biostimulant positively impacted the maize plant by aiding in maintaining or improving plant health. Seaweed extract-based biostimulants were reported to upregulate the levels of phenylpropanoids in tomato plants and “Sangiovese” grapes [11,51]. Strawberry plants treated with seaweed extract-based biostimulants showed enhanced levels of carbohydrates and quercetin [23]. Furthermore, abscisic acid, cytokinin, and indole acetic acid levels were elevated in Arabidopsis thaliana plants treated with seaweed-based biostimulants [7]. Application of these various seaweed extract-based biostimulants were shown to enhance nitrogen, protein, lipid, polyphenolic (e.g., flavonoids), tannic, and sugar contents in maize plants [52,53]. Additionally, the efficacy of seaweed extract-based biostimulants on improving stress resistance in maize plants was reported [54]. For instance, the foliar application of *Kappaphycus alvarezii* seaweed extract was proven effective in ameliorating drought stress in maize, through the enhancement of antioxidants and changes in physiological processes [55].

### 2.2. Impacted Biological Pathways and Changes in Plant Height and Diameter of Maize Plants Treated with a Seaweed-Based Biostimulant

**Figure 8:**
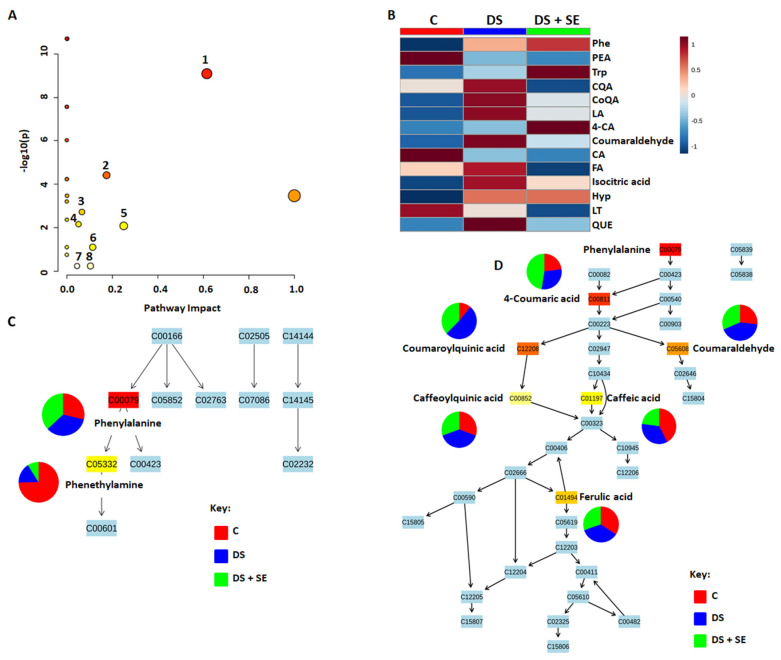
Summary of Metabolic Pathway Analysis (MetPA) outputs. (**A**) Representation of the global metabolome overview containing all of the matched pathways arranged by *p*-Values on the *y*-axis and pathway impact (significance) values on the *x*-axis. The pathway impact values refer to the cumulative percentage from the matched metabolites and the maximum importance of each pathway is 1. (1) Phenylalanine metabolism; (2) Phenylpropanoid biosynthesis; (3) Arginine and proline metabolism; (4) Flavonoid biosynthesis; (5) Stilbenoid, diarylheptanoid, and gingerol biosynthesis; (6) alpha-Linolenic acid metabolism; (7) Citrate cycle (TCA cycle); and (8) Glyoxylate and dicarboxylate metabolism; (**B**) Heatmaps displaying differential qualitative alterations in the concentrations of selected metabolites; (**C**) Topological characteristics of the phenylalanine metabolism pathway, including quantification levels of phenylalanine and phenethylamine; (**D**) Topological characteristics of the phenylpropanoid biosynthesis pathway including quantification levels of 4-coumaric acid, p-coumaroylquinic acid, caffeoylquinic acid, p-coumaraldehyde, caffeic acid, and ferulic acid. Abbreviation: C, Control (no stress; no seaweed extract biostimulant); DS, Drought stress (stress; no seaweed extract biostimulant); DS + SE, Drought stress with seaweed extract biostimulant; Phe, Phenylalanine; PEA, Phenethylamine; Trp, Tryptophan; CQA, Caffeoylquinic acid; CoQa, Coumaroylquinic acid; LA, Linolenic acid; 4-CA, 4-Coumaric acid; CA, Caffeic acid; FA, Ferulic acid; Hyp, 4-hydroxyproline; LT, Luteolin; and QUE, Quercetin.

To situate the maize metabolic changes upon biostimulant application (as described in Section 2.1) in a biological context, metabolic pathway analysis was performed. Based on quantitative pathway analysis of the differentially abundant metabolites due to seaweed treatment, we observed specific patterns per control/treatment group for metabolites in the phenylalanine metabolism, phenylpropanoid biosynthesis, and flavonoid biosynthesis pathways (Figure 8A and Table S4). The metabolites involved in these pathways, under drought stress conditions, were observed to be altered by the seaweed-based biostimulant treatment, resulting in specific metabolite patterns (Figure 8B).

The seaweed-based biostimulant treatments on the maize plants under drought stress conditions induced differential alteration on the phenylalanine metabolism, with an increase in phenylalanine and significant decrease in phenethylamine observed (Figure 8C). The soil method application of the seaweed-based biostimulant had the most positive effect on the phenylalanine metabolite, whereas for phenethylamine, a negative effect was observed for the seaweed-based biostimulant treatment (Figure S5A). The increased phenylalanine levels in the maize plants is due to the effect of the seaweed biostimulant, which was reported to induce the activity of phenylalanine ammonia lyase [7]. The increased phenylalanine levels are indicative of the induction of the phenylpropanoid pathway, an epicenter of defense-related metabolites [10]. Phenylalanine is thus regarded as a key metabolite that serves as a bridge between the phenylalanine metabolism to other specialized metabolic pathways, as it serves as a precursor of secondary metabolites, such as flavonoids, alkaloids, and phenylpropanoids, and as a stress-related signal [56,57,58].

The seaweed-based biostimulant induced reprogramming of the phenylpropanoid biosynthesis pathway, as observed by the changes in the matched metabolites that include phenylalanine, coumaric acid, caffeic acid, ferulic acid, coumaraldehyde, and chlorogenic acids (i.e., coumaroylquinic acid and caffeoylquinic acid) (Figure 8D). The coumaric acid and coumaraldehyde levels were evidently increased in the seaweed-based biostimulant-treated plants under drought stress conditions, with the foliar application method having the more prominent increase in coumaric acid levels over the soil application method (Figure 8D and Figure S4B). Coumaric acid is one of the main constituents of coumarins and phenylpropanoids, which actively function in physiological processes, plant adaptation, and resistance/tolerance to mechanical and biological stresses. Coumaric acid functions as a signaling molecule for plant development at different stages and its increase in concentration levels is suggestive of changes induced by the seaweed-based biostimulant in the maize plants’ physiological processes towards drought stress tolerance [59,60]. Similar to coumaric acid levels, tryptophan levels were also notably increased in seaweed-based biostimulant treated plants under drought stress conditions (Figure 8B). Increased levels of tryptophan in maize plants under drought stress conditions were reported and suggested to be attributed to its function as a precursor for a range of specialized metabolites and its role in scavenging ROS [61].

Coumaraldehyde is a key precursor for the biosynthesis of lignins that function in cell wall strengthening activities under stress conditions [40,62]. The accumulation of coumaraldehyde can therefore be related to cell wall strengthening, and thus the results suggest the seaweed-based biostimulant may function in promoting cell wall strengthening through enhanced biosynthesis of lignins. Seaweed-based biostimulants were reported to increase the accumulation of lignins and stimulate pathways associated with lignin biosynthesis in oilseed rape and bean plants [8,63]. Furthermore, the accumulated levels of coumaraldehyde, suggest that the applied biostimulant may play a role in maintaining the strength of the plant cell wall under drought stress conditions.

Furthermore, variations in chlorogenic acids (i.e., coumaroylquinic and caffeoylquinic acids) levels were observed. The coumaroylquinic acid levels were increased in the seaweed-based biostimulant treated maize plants under drought stress conditions, with the foliar application method having the positive effect (Figure 8D and Figure S5B). No notable changes were observed in caffeoylquinic acid levels; however, according to the heatmap analysis, the levels were increased for both foliar- and soil-applied biostimulant treatment. The soil application method is shown to elicit the greatest increase in the caffeoylquinic acid levels (Figure 8D and Figure S5B). These chlorogenic acids are reported to have antioxidant activities and their increase in maize leaves during the onset of stress, are shown to negatively reduce the plants’ growth and yield-related traits [27].

Differential effects of the seaweed-based biostimulant on drought-stressed maize plants’ height and diameter were observed. The maize plants treated with the foliar application of the seaweed-based biostimulant were observed to be significantly taller and thicker than the untreated plants, whereas those treated with the soil application displayed no significant effect on the plant height and diameter (Tables S5 and S6, Supplementary Materials). On the contrary, under normal conditions, the maize plants treated with the soil application of the seaweed-based biostimulant were observed to be taller and thicker than the untreated plants, and those treated with the foliar application displayed no significant effect on the plant height and diameter (Tables S5 and S6, Supplementary Materials). Drought stress was reported to negatively affect plant growth parameters, such as plant height and stem diameter, therefore the increase in plant height and diameter during the onset of drought is suggestive of enhanced plant tolerance to stress [64]. Weekly foliar application of pot-grown maize seedlings with seaweed extracts for 90 days were reported to have a significant effect on the plant diameter and resulted in an increase in plant length by an average of 43% in comparison to control seedlings [65]. It was reported that during growth, plants accumulate sugars, phytohormones, coumaroyl- and feruloyl-related metabolites in the roots, whereas, when stressed, regulatory and defense-related metabolites, such as antioxidants, abscisic acid, coumaroyl- and feruloyl-related metabolites, may be synthesized or act in specific plant tissues [66,67]. Thus, the observed increase in coumaroylquinic acid levels in the foliar application seaweed-based biostimulant treated plants under drought stress (Figure S5B, Supplementary Materials), may be related to the increase in the plant’s height and diameter. Whereas in the soil application seaweed-based biostimulant treated plants under normal conditions, the growth in plant height and diameter can be related to the seaweed-based biostimulant’s growth promoting substances, which were reported to include phytohormones, sugars, and phenolics [54]. It can also be suggested that the seaweed-based biostimulant plays a role stress tolerance during unfavorable environmental conditions and growth promotion during favorable environmental conditions.

The caffeic acid levels were decreased, whereas the ferulic acid levels showed no significant changes in the seaweed-based biostimulant treated plants under drought stress conditions (Figure 8D and Figure S5B). The heatmap for ferulic acid, however, depicts a decrease in its levels, and for caffeic acid, the soil application method showed a positive effect on its levels (Figure S5B). Both caffeic and ferulic acids, in the same way as other phenolic metabolites, are primarily involved in lignin synthesis and cell wall formation [68,69]. Additionally, caffeic acid is also involved in the regulation of turgor pressure, water flux, and growth, thus describing its importance in maintaining plant growth and tolerance to stress, as reflected by the observed caffeic acid levels in the maize leave samples’ drought stress conditions [68]. Ferulic acid levels were reported to be decreased in maize under drought stress conditions and the observed results are indicative that the biostimulant does not favor the increase in ferulic acid drought stress conditions [40]. Considering that some of these phenylpropanoid pathway products serve as precursors for other pathways, the stimulation of this pathway by the seaweed-based biostimulant may therefore impact other downstream pathways, such as the flavonoid pathway [70].

The impact of the seaweed-based biostimulant treatment on the flavonoid biosynthesis pathway is observed by the changes in the matched metabolites, that include luteolin, quercetin, and the phenylpropanoid products, coumaroylquinic and caffeoylquinic acid (Figure S6A). The biostimulant resulted in a decrease in luteolin and quercetin levels under drought stress conditions (Figure S6A). The heatmap for quercetin, however, shows an increase in its levels with the soil application method of the biostimulant treatment, whereas for luteolin, both methods of application had negative effects on its accumulation (Figure S6B). Luteolin and quercetin are reported to be the most powerful antioxidants among the flavonoids and are associated with plant developmental regulation and stress response/tolerance [67,71]. Both luteolin and quercetin are reported to be exudates in seaweed extracts [23]. Quercetin and their derivatives were proved to improve water and nutrient uptake of plants through interaction with the soil chemistry and enhancement of the lateral root formation. In addition, they regulate auxin transport and the abscisic acid signaling pathways by antagonizing the abscisic acid-regulated stomatal closure through the reduction of oxidative stress levels, thereby enabling the plant to respond to stress in a more conservative manner [37,67]. Quercetin levels were suggested to be decreased due to their glycosylation and use in the synthesis of other flavonoids in defense to salt stress, hence this may possibly explain the observed quercetin and luteolin levels [72]. Furthermore, in the study of [73], a seaweed extract-based biostimulant was reported to enhance the plant production of quercetin, as similarly observed in our study (Figure S6B, Supplementary Materials).

**Figure 9:**
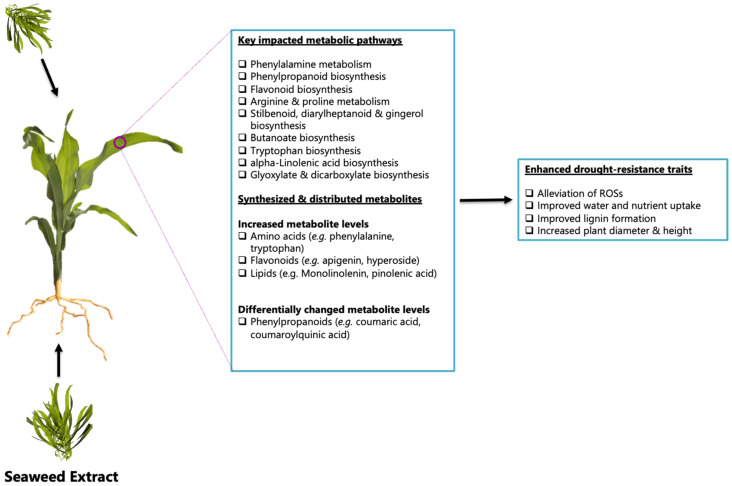
A summary of the results obtained in this study highlighting the general seaweed-based biostimulant’s mechanism of action. The metabolic and biochemical events observed in the seaweed-based biostimulant treated maize plants via soil and foliar application, displayed alterations in the primary and specialized metabolic pathways that resulted in increased synthesis and distribution of metabolites related to enhanced drought resistance traits.

Overall, the observed metabolic alterations suggest that the application of the seaweed-based biostimulant cautiously facilitated the maize tolerance to drought stress without vastly compromising its growth and development, by increasing the accumulation of metabolites associated with antioxidant activities, plant physiological signaling, and the biosynthesis of secondary metabolites (Figure 9). Tryptophan is one example that was widely accumulated in the leaves, as it serves as a precursor for a range of metabolites including lignin precursors, and, as suggested, it also serves as a protein protectant from ROS [61]. Additionally, based on the metabolite levels and comparison of the methods of application, it can be suggested that the method of application plays a role in the accumulation of specific metabolites and thereby the reconfiguration of specific maize metabolic pathways. The soil application method was shown to have the greatest impact on the maize metabolic pathways. For instance, caffeic acid and related metabolites in the phenylpropanoid pathway, as well as metabolites in the phenylalanine and flavonoid pathways, were greatly increased with the soil application method in comparison to the foliar application method. However, the foliar application method had a more positive effect on the distributions of coumaroylquinic acid and coumaraldehyde metabolites. This phenomenon may be attributed to the tissue-specific distribution of the metabolites and therefore suggests that the application method may determine which molecular mechanisms/events are prioritized, i.e., the increase in antioxidant activity over plant growth and development, or vice versa. Thus, the soil application method can be considered best for drought stress conditions, as it facilitates the increase in most metabolites involved in both defense or tolerance responses, plant growth, and development. In future, the combination of metabolomics’ and phenomics’ studies on the root tissues may provide insight into the maize plant’s uptake of seaweed-based biostimulant exudates and their mechanisms of action in drought-stressed plants.

The generalized biostimulant mechanisms of action were found to include the reconfiguration of primary and specialized metabolic pathways, such as the phenylalanine, phenylpropanoid, flavonoid, and fatty acid pathways (Figure 9). The reconfiguration of these pathways encompassed the increased synthesis and/or distribution of metabolites previously reported to be involved in plant physiological signaling and homeostasis, oxidative stress alleviation, cell wall formation and strengthening, water and nutrient uptake, and lateral root formation (Figure 9). Nevertheless, additional studies, such as the analysis of root and shoot metabolites of maize plants treated with seaweed-based biostimulants, are required to yield further insight and prove the effect of the seaweed-based biostimulant on the reported physiological events.

## 3. Materials and Methods

All chemicals for sample analyses (from the pre-analytical step to the data acquisition stage) were of analytical or pure grade quality and obtained from various international suppliers. Briefly, the organic solvents used, methanol and acetonitrile, were LCMS grade quality (Romil, MicroSep, South Africa). Water was purified by a Milli-Q Gradient A10 system (Millipore, Billerica, MA, USA). Leucine enkephalin and formic acid were purchased from Sigma Aldrich, Steinheim am Albuch, Germany. The study design and plants’ cultivation are detailed in the following section.

### 3.1. Maize Plant Preparation, Cultivation and Phenotypic Measurements

The maize (*Zea mays*) plants, PAN 3Q-240, were cultivated in 10 L-pots, containing a sandy soil, placed in a randomized order on rotating tables in a greenhouse at Omnia facilities in Sasolburg, Free-State, South Africa. Drought stress, the drop in water levels to below 50% plant available water (PAW), was applied at the 3-leaf stage (3 weeks after emergence). The water level was dropped and maintained at 50% plant available water (PAW). Well-watered plants were maintained at 90% PAW. Details on how the 50% PAW and 90% PAW were determined are highlighted in the Supplementary Materials (Section S3.1). The study was experimentally designed to comprise control and treated groups, all referred to as treatments (Table S7, Supplementary Materials). Control groups consisted of (1) control (no seaweed extract, no drought stress); (2) control (no seaweed extract with drought stress); treated groups were (3) soil applied seaweed extract with no drought stress; (4) soil applied seaweed extract with drought stress; (5) foliar applied seaweed extract with no drought stress; and (6) foliar applied seaweed extract with drought stress (Table S7, Supplementary Materials). The biological changes reflecting the seaweed extract-treated and naïve plant responses to drought stress conditions were monitored over 1-, 3-, 7- and 14-days post treatment, i.e., after drought application. Each pot was considered a biological replicate and contained five plants at the harvesting time. Five biological replicates (i.e., five pots) per group (per treatment, Table S7, Supplementary Materials) were harvested at each time point. The seaweed extract used in this study is a Kelpak^®^ formulation, made from kelp *Ecklonia maxima* and registered as a biostimulant for use in agriculture. For soil treatment, the seaweed extract formulation was added to the soil. The foliar treatment was applied by using a pressure spray and evenly applying the formulation on maize leaves at the 4-leaf stage (4 weeks after emergence).

The plant height was measured with a ruler from the soil surface (base) to the collar of the most recently unfolded leaf. The plant stem diameter was measured with a caliper.

The plant leaves were cut off at the node, frozen with liquid nitrogen and stored at −80 °C prior to metabolite extraction.

### 3.2. Metabolite Extraction and Sample Preparation

Metabolites were extracted from treated and non-treated plant leaves using 80% cold aqueous methanol, in a ratio of 1:15 (*w*/*v*), at 4 °C. The mixture was homogenized using an Ultra Turrax homogenizer, followed by sonication using a probe sonicator (Bandelin Sonopuls, Germany), set at 55% power for 15 sec. The homogenates were centrifuged at 5000× *g* for 10 min at 4 °C, and supernatants were kept. The 80% aqueous-methanol extracts were concentrated by evaporating the supernatants to complete dryness and re-suspending the dried extracts in 300 µL 50% aqueous-methanol. The samples were then filtered through 0.22 µm nylon syringe filters (Anatech, Randburg, South Africa) into HPLC glass vials fitted with 500 µL inserts. The filtered extracts were kept at −20 °C until analyzed. The methanol used was LC-grade (Romil Pure Chemistry, Cambridge, UK) and ultrapure water (Siemens purification system, Separations, Randburg, South Africa). The quality control (QC) samples were pooled samples, prepared by pipetting and mixing aliquots of equal volume from all of the samples.

### 3.3. Sample Analyses on an UHPLC-HDMS Analytical Platform

Sample analyses were carried out on a Waters Acquity ultra-high performance liquid chromatography (UHPLC) system coupled to a SYNAPT G1 Q-TOF mass spectrometer equipped with an electrospray ionization (ESI) source (Waters Corporation, Milford, CT, USA). A Waters HSS T3 C18 column (150 mm × 2.1 mm ×1.8 µm) thermostatted at 60 °C was used for the chromatographic separation of the samples, with an injection volume of 2 µL. The mobile phase was a binary solvent system consisting of 0.1% aqueous formic acid (Sigma-Aldrich, Darmstadt, Germany) (solvent A), and 0.1% formic acid in acetonitrile (Romil Pure Chemistry, Cambridge, UK) (solvent B), at a flow rate of 0.4 mL min^−1^. The initial gradient elution conditions were 98% A and 2% B, maintained for 13 min followed by 30% A and 70% B at 14 min; at 15 min the conditions were changed to 5% A and 95% B for 2 min, followed by a return to the initial conditions. The column was calibrated for 2 min prior to the next injection. The total chromatographic run time was 20 min. Individual samples were analyzed in triplicate to account for any analytical variability.

For mass spectrometry (MS) analyses, both ESI positive and negative modes were used, and data were collected covering the 100–1000 Da mass range. Other MS conditions were set as follows: capillary and sampling cone voltages, 2.5 kV and 30 V, respectively; extraction cone voltage, 4.0 V; source temperature, 120 °C; desolvation temperature, 450 °C; cone gas flow, 50 L h^−1^ and desolvation gas flow, 550 L h^−1^. The scan time was 0.2 s. Nitrogen was used as the nebulization gas with a flow rate of 700 L h^−1^. Fragmentation data were obtained using a data independent acquisition (DDA) method, MS^E^, with collision energy ramping from 10 to 30 eV. Leucine encephalin (50 pg mL−1), [M + H]^+^ = 55.2766 and [M–H]^−^ = 554.2615, was used as a lock spray mass, to ensure high mass accuracy between 1–3 mDa and reproducibility. The software used to control the hyphenated system and perform all of the data manipulation was MassLynxTM 4.1 (SCN 704, Waters Corporation, Milford, CT, USA).

Solvent blanks and the quality control (QC, pooled) samples were also analyzed in parallel with the sample extracts. The blank samples (50% aqueous methanol) were randomly run to monitor for background noise. The QC samples were used to condition the LC–MS analytical system and to assess the reliability and reproducibility of the analysis [74,75,76,77]. The samples were analyzed in a randomized manner, with the QC sample analyzed every 10 injections to monitor and correct changes in the instrument response.

### 3.4. Data Analysis: Data Set Matrix Creation and Chemometric Analyses

Visualization and data processing were performed using MassLynx XSTM 4.1 software (Waters Corporation, Manchester, UK). The MarkerLynx^TM^ application manager of the MassLynx software was used for data pre-processing (matrix creation), producing a matrix of retention time (Rt)-*m*/*z* variable pairs, with *m*/*z* peak intensity for each sample. MarkerLynx software parameters were set to process the 1–15 min Rt range of the chromatograms and the *m*/*z* domain of mass range 100–1000 Da. The Rts were allowed to differ by ± 0.2 min and the *m*/*z* values by ±0.05 Da. The mass tolerance used was 0.01 Da, and the intensity threshold was 100 counts. Only data matrices that had a noise level of less than 50% (MarkerLynx cut off) were retained for downstream chemometrics and statistical analyses.

### 3.5. Molecular Networking in GNPS

The format of the raw Waters MS/MS data was converted to the analysis base file (ABF) format using the Reifys Abf converter software (https://www.reifycs.com/AbfConverter/, accessed on 22 May 2022), and then uploaded onto the Mass Spectrometry-Data Independent AnaLysis (MS-DIAL) software for data-processing. The MS-DIAL parameters used to process the data were a mass accuracy (MS1 and MS2 tolerance) of 0.05 Da, minimum peak height of 10 amplitude and mass slice width of 0.1 Da for peak detection, a 0.5 sigma window value, and a 0 MS/MS abundance cut-off for data deconvolution; a retention time tolerance of 0.05 min was used under alignment parameter settings, with one of the QC samples used as a reference file for alignment. Following data-processing with MS-DIAL, the resultant GNPS export files, i.e., GNPS MGF and GNPS Table (feature quantification table) were then uploaded onto the GNPS server (https://gnps.ucsd.edu/, accessed on 22 May 2022) using the WinSCP server software.

The FBMN workflow was applied for data acquired in both the negative and positive electrospray ionization modes, by uploading the respective feature quantification table, MGF file, and metadata file describing the properties of the sample file (i.e., treatment, day, and plant condition). The parameters used to generate the FBMN were a precursor ion mass tolerance of 0.05 Da, a fragment ion mass tolerance of 0.05 Da, a minimum pair cosine score of at least 0.7 with a minimum of six matched peaks, and the search analogs was turned off. The resultant molecular networks were further analyzed with MS2LDA, NAP, and MolNetEnhancer (all accessible through the GNPS ecosystem). The parameters for MS2LDA were set as follow: bin width of 0.01, number of iterations at 1000, 300 free motifs, and a minimum MS2 intensity of 100. The MotifSets selected from the MotifDB included GNPS, MassBank, Euphorbia, Streptomyces and Salinisporus, Photorhabdus and Xenorhabdus (for positive ionization), and Rhamnaceae Plant (for negative ionization). The discovered Mass2Motifs or substructures were analyzed within the MS2LDA.org web application and mapped on the nodes of the mass spectral molecular networks, with the shared Mass2Motifs among the various nodes mapped on the edges connecting the nodes. NAP utilized the MetFrag in silico fragmentation tool to search the structural databases of GNPS, Dictionary of Natural Products (DNP), Super Natural II (SUPNAT), Chemical Entities of Biological Interest (ChEBI), and MarinLit [78]. The accuracy for exact mass candidate search was set to 10 ppm. Fusion and Consensus scores were calculated based on the 10-first candidates in the network propagation phase.

The MolNetEnhancer workflow enhanced the molecular networks by combining the outputs of FBMN, MS2LDA, and NAP to improve the chemical structural annotations. The resultant FBMN, MS2LDA, and MolNetEnhancer molecular networks were visualized using the Cytoscape version 3.7.2 tool/software [79,80]. The fragmentation spectra of all of the putatively annotated metabolites matched to the GNPS’ spectral libraries were manually validated, using the metabolite annotation workflow described below.

### 3.6. Metabolite Identification and Metabolic Pathway Analyses

#### 3.6.1. Metabolite Identification

For metabolite identification, the data matrices from MarkerLynx-based data processing were exported to the Taverna workbench for PUTMEDID_LCMS Metabolite ID Workflows [81,82]. The Taverna workflows allow for integrated, automated, and high-throughput annotation and putative metabolite identification from LC–ESI-MS metabolomic data. The workflows consist of correlation analysis, metabolic feature annotation, and metabolite annotation. A data matrix from MarkerLynx-based data processing was firstly formatted to match the Taverna workbench requirements. Three main workflows formed the Taverna Metabolite ID procedure: (I) Pearson-based correlation analysis (List_CorrData); (II) metabolic feature annotation (annotate_Massmatch)—allowing for grouping together ion peaks with similar features, such as Rt, and annotating features with the type of *m*/*z* ion (molecular ion, isotope, adduct, others) believed to originate from the same compound. The elemental composition molecular formula (MF) of each *m*/*z* ion is then automatically calculated; and (III) metabolite annotation (matchMF-MF) of the calculated MF (from the output file from workflow (2) is automatically compared and matched to the MF from a pre-defined reference file of metabolites.

Three main steps were carried out for annotation confidence: (i) the calculated MF of a selected metabolite candidate was manually searched against databases and bioinformatics tools (mainly, DNP, Chemspider, SorgCyc, PlantCyc, and KEGG); (ii) structural confirmation through careful inspection of fragmentation patterns by examining the MS1 and MSE spectra of the selected metabolite candidate; (iii) comparative assessment with/against annotation details of metabolites in sorghum, reported in literature, particularly in [83,84].

In this study, during substructure mining, we focused on the previously annotated Mass2Motifs from MotifDB MotifSets derived from GNPS, MassBank, Euphorbia, Rhamnaceae Plant (only for negative mode ionization), Streptomyces and Salinisporus, Photorhabdus and Xenorhabdus MotifSets that were all matched to MS/MS spectra of metabolite features in the maize molecular networks. The annotated GNPS spectral library matches were subsequently used to aid in structural identification of the mass peaks and molecules matching the in MotifDB annotated Mass2Motifs.

#### 3.6.2. Pathway Analyses

Pathway analysis of annotated metabolites were performed with the MetPA (Metabolomics Pathway Analysis) component of the MetaboAnalyst bioinformatics tool suite (version 3.0; http://www.metaboanalyst.ca/, accessed on 22 May 2022) [20,21], enabling the identification of the affected metabolic pathways, analysis thereof, and visualization. MetPA uses high-quality KEGG metabolic pathways as the backend knowledge base.

## 4. Conclusions

Metabolomics and molecular networking computational strategies provided key insights into the mechanisms of action of seaweed extract-based biostimulants through the wide-screen exploration of the maize metabolome under drought stress conditions. The molecular networking strategies enabled the characterization of the reconfigured maize chemical space, and pathway analysis provided insight on the impacted pathways due to the seaweed-based biostimulant treatment. The phenylalanine, phenylpropanoid, flavonoid, and fatty acid biosynthesis pathways were some of the primary and specialized metabolic pathways reconfigured by the seaweed-based biostimulant. These metabolic reconfigurations increased the maize plant height and diameter under normal and drought stress conditions, with the foliar application method having the most positive effect on the plants subjected to drought stress, whereas under normal conditions, the soil application method increased the plant height and diameter. Furthermore, the changes in metabolite profiles attributed to the seaweed-based biostimulant treatment and the method of application were associated with previously reported metabolites involved in stress-related responses. This knowledge provided about the underlying mechanisms of seaweed extracts-based biostimulants on maize plants under drought stress will potentially increase widespread application and science-based development of biostimulants.

## Data Availability

The study LC-MS data, GNPS MGF, GNPS Table, and metadata can be found on https://massive.ucsd.edu/, MSV000089083 (positive ionization), and MSV000089084 (negative ionization).

## Supplementary Materials

The following supporting information can be downloaded at: https://www.mdpi.com/article/10.3390/metabo12060487/s1, the GNPS job links; Figure S1: Principal component analyses (PCA) score plots; Figure S2: Feature-based molecular network (FBMN) of positive electrospray ionization (ESI+) MS/MS spectra; Figure S3: MolNetEnhancer enriched feature-based molecular networks; Figure S4: A pie chart of selected differential metabolite features; Figure S5: Heatmap analyses of qualitative altered metabolites; Figure S6: Summary of Metabolomics Pathway Analysis (MetPA) outputs; Table S1: Annotation of unique metabolites based on the spectral matches of experimental data with the GNPS library databases; Table S2: Metabolites manually annotated; Table S3: Discriminating features (*m*/*z* ions) selected using OPLS-DA modelling; Table S4: Significantly altered metabolic pathways; Table S5: Height analyses of maize plants with and without seaweed-based biostimulant treatment; Table S6: Diameter analyses of maize plants with and without seaweed-based biostimulant treatment; Table S7: Seaweed extract biostimulant application rates (L/ha) applied to the plant sample groups.
