# Supplementary material for: Computational Metabolomics Tools Reveal Metabolic Reconfigurations Underlying the Effects of Biostimulant Seaweed Extracts on Maize Plants under Drought Stress Conditions
Source: Metabolites. 2022 May 27;12(6):487. doi: 10.3390/metabo12060487 (PMC9231236; doi:10.3390/metabo12060487)
Supplement: Supplementary file 1 [file metabolites-12-00487-s001.zip › metabolites-1666854-supplementary.pdf]

Supplementary File

Computational metabolomics tools reveal metabolic reconfigurations underlying the effects of biostimulant seaweed extracts on maize plants under drought stress conditions

Morena M. Tinte[1], Keabetswe Masike[1], Paul A. Steenkamp[1], Johan Huyser[2], Justin J.J. van der Hooft[3*] and Fidele Tugizimana[1,2*]

Section S3.1: Determination of Plant available water (PAW)

The soil field capacity (FC) and permanent wilting point (PWP) were determined in the following manner. A pot was filled with soil and flooded with water. The pot was then sealed on top with cling wrap and the water was left to drain at the bottom of the pot for 3 days. The wet soil was then weighed and the mass was regarded as the FC. Thereafter, the soil was dried in an oven at 50°C and weighed, the mass was considered as the PWP. The plant available water (PAW) was then determined as follows:

(i) PAW = FC – PWP
(ii) 90% PAW = (PAW × 0.9) + PWP
(iii) 50% PAW = (PAW × 0.5) + PWP

GNPS Results

GNPS job links:

1. Negative electrospray ionization mode:

   FBMN: https://gnps.ucsd.edu/ProteoSAFe/status.jsp?task=9a78bc6fc12648b6b6a0917990748f1f
   MS2LDA:
   https://proteomics2.ucsd.edu/ProteoSAFe/status.jsp?task=da6156bee08947b2b3b6e100ce918aa9
   NAP: https://proteomics2.ucsd.edu/ProteoSAFe/status.jsp?task=af3fcc8836b446bfa9772d1e69b54dce
   MolNetEnhancer:
   https://gnps.ucsd.edu/ProteoSAFe/status.jsp?task=6430f5896eab45d2b40819a52e008e54

2. Positive electrospray ionization mode:

   FBMN: https://gnps.ucsd.edu/ProteoSAFe/status.jsp?task=3acba582a78a40dd8618d9cfc1c0ea16
   MS2LDA:
   https://proteomics2.ucsd.edu/ProteoSAFe/status.jsp?task=6a1178a2a5c74127880679fca96f31a1
   NAP: https://proteomics2.ucsd.edu/ProteoSAFe/status.jsp?task=9df38400bd244088a406a1a476901a50
   MolNetEnhancer:
   https://gnps.ucsd.edu/ProteoSAFe/status.jsp?task=6500bf69794e4fdf91532f97e1a8328e

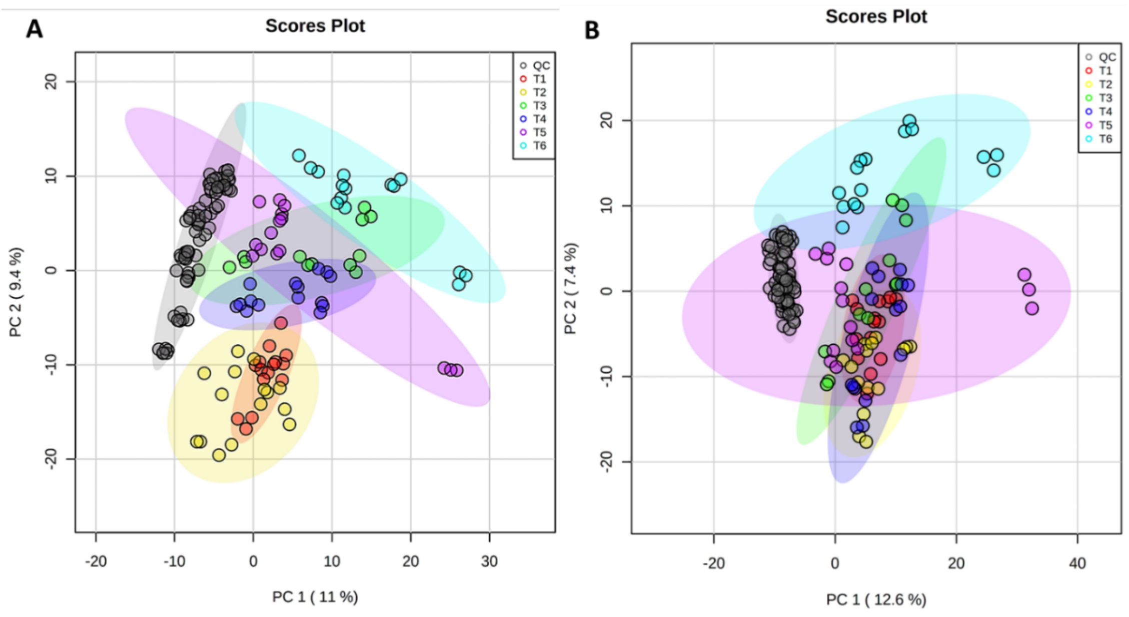

Figure S1. Principal component analyses (PCA) score plots for (**A**) electrospray ionisation (ESI) positive (ESI^+) data that explains 42.7% variation in pareto-scaled data with 2.6% predictive variation, and (**B**) ESI negative (ESI^-) data explaining 41.3% variation in pareto-scaled data with 2.2% predictive variation. The sample groupings are coloured according to stress conditions, biostimulant treatment and method of application (QC = black; T1 = red; T2 = yellow; T3 = green; T4 = blue; T5 = purple; and T6 = cyan). **Abbreviations/key:** QC, quality control; T1, control 1 (no biostimulant application under normal conditions); T2, control 2 (no biostimulant application under drought stress conditions); T3, soil applied biostimulant under normal conditions; T4, soil applied biostimulant under drought stress conditions; T5, foliar applied biostimulant under normal conditions; and T6, foliar applied biostimulant under drought stress conditions.

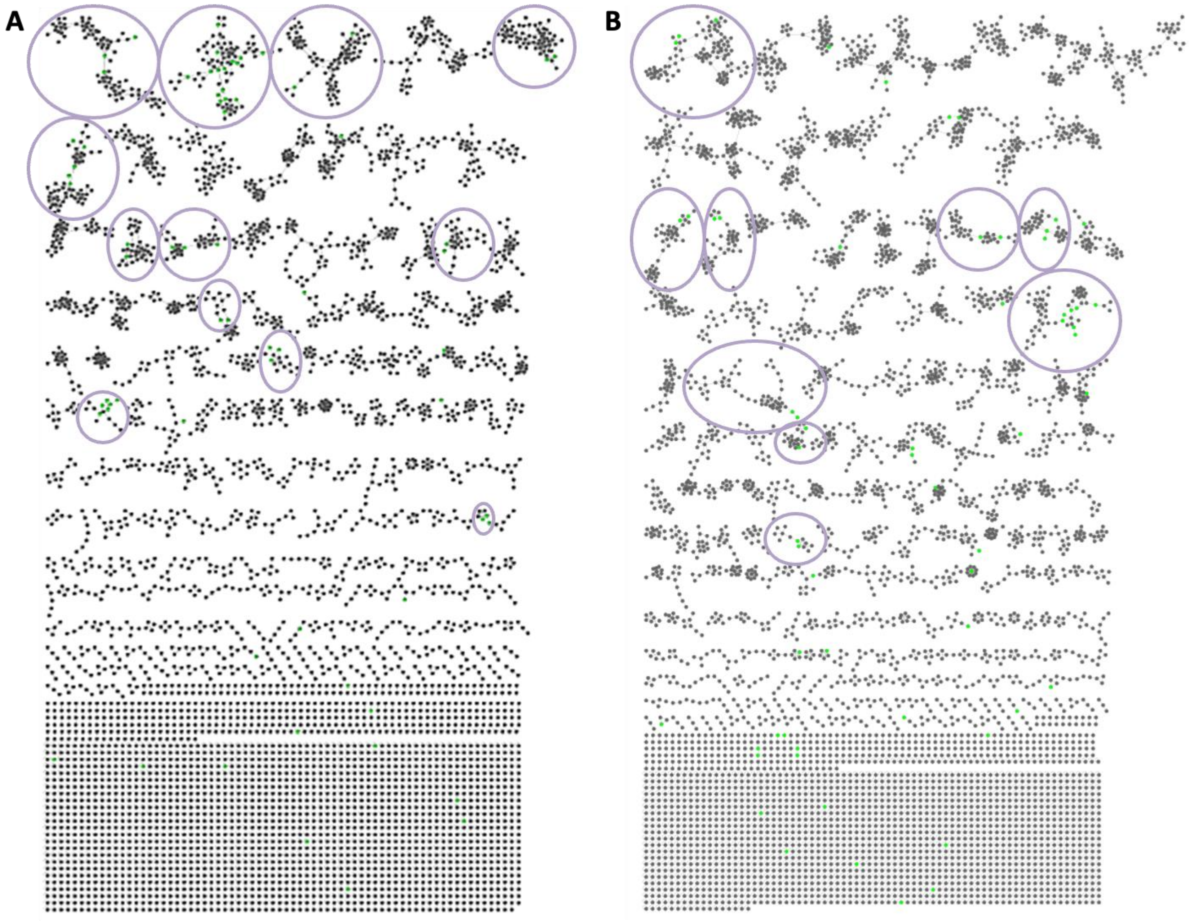

Figure S2. Feature-based molecular network (FBMN) of (**A**) positive electrospray ionisation (ESI^+) and (**B**) negative electrospray ionisation (ESI^-) MS/MS spectra obtained from maize leave extracts. The molecular networks depict structurally similar nodes as molecular families/clusters, with the nodes which mass spectra had a spectral match in the GNPS public mass spectral libraries represented by green coloured nodes and the unknowns by grey coloured nodes. The molecular families encircled have two or more annotated nodes, and these highlight the (better) known chemistry within the data.

Table S1. Annotation of unique metabolites based on the spectral matches of experimental data with the GNPS library databases with the conditions of a 0.7 cosine score threshold and a minimum of 6 shared mass peaks between query and library mass spectrum.

Metabolite Annotation	Metabolite Abbreviation	Precursor mass (*m/z*)	Library mass (*m/z*)	Diagnostic fragments (*m/z*)	Score Threshold	Ionisation Mode	ClassyFire Class Annotation	Manually assigned Chemical Class Terms
4-aminobutanoate		104.11	104.11	60	0.7	Positive	Carboxylic acids and derivatives	Amino acids and derivatives
Phenethylamine	PEA	122.10	122.10	105	0.7	Positive	No match	Benzene and substituted derivatives
4-hydroxyproline	Hyp	132.11	132.07	114; 86	0.7	Positive	No match	Amino acids and derivatives
Adenine	Ade	136.06	136.06		0.7	Positive	Carboxylic acids and derivatives	Imidazopyrimidines
1-Naphthol	1-NAP	145.03	145.06	117; 115; 103	0.7	Positive	Organic compounds	Organic compounds
2-Naphthol	2-NAP	145.03	145.06	117	0.7	Positive	Organic compounds	Organic compounds
5,6 Dimethylbenzimidazole		147.04	147.09	132	0.7	Positive	Carboxylic acids and derivatives	Benzimidazoles
p-Coumaraldehyde		147.05	147.04		0.7	Positive	Cinnamaldehydes	Cinnamaldehydes
(4S,5Z,6S)-4-(2-methoxy-2-oxoethyl)-5-[2-[(E)-3-phenylprop-2-enoyl]oxyethylidene]-6-[(2S,3R,4S,5S,6R)-3,4,5-trihydroxy-6-(hydroxymethyl)oxan-2-yl]oxy-4H-pyran-3-carboxylic acid		149.03	149.06	121	0.7	Positive	Phenols	Organooxygen compounds
Rosmarinic acid	RosA	163.04	163.04	145; 135; 117; 107; 89	0.7	Positive	Carboxylic acids and derivatives	Cinnamic acids and derivatives
5-Methyl-2-benzofuran-1,3-dione		163.05	163.04	135; 133	0.7	Positive	Carboxylic acids and derivatives	Benzofurans
4-Coumaric acid	4-CA	165.06	165.06	147; 119; 91	0.7	Positive	Carboxylic acids and derivatives	Cinnamic acids and derivatives
Ferulic acid	FA	177.06	177.05	163; 149; 145;	0.7	Positive	Carboxylic acids	Cinnamic acids and

				135; 117; 89			and derivatives	derivatives
Caffeic acid	CA	181.06	181.04	163; 145; 135; 117	0.7	Positive	Carboxylic acids and derivatives	Cinnamic acids and derivatives
3-Methyldiphenylamine		184.08	184.11		0.7	Positive	Carboxylic acids and derivatives	Pyridines and derivatives
3,4-Methylenedioxy-N-methylamphetamine	MDMA	194.12	194.12	163; 135	0.7	Positive	Carboxylic acids and derivatives	Benzodioxoles
3-Hydroxy-4-methoxycinnamic acid	Isoferulic acid	195.07	195.07	177; 163; 149; 145; 135; 117; 89	0.7	Positive	Carboxylic acids and derivatives	Cinnamic acids and derivatives
3-Nitro-L-tyrosine	3-Nitrotyrosine	225.04	225.05	181; 163; 145; 135	0.7	Positive	Carboxylic acids and derivatives	Carboxylic acids and derivatives
Pinolenic acid	PA	261.22	261.22	243; 233; 219; 215; 187; 177; 173; 163; 159; 149; 145; 135; 131; 121; 109; 107; 93; 81; 67	0.7	Positive	Lipids	Fatty Acyls
Oleamide		282.28	282.27	265; 247; 212; 184; 163; 156; 149; 135; 121	0.7	Positive	Lipids	Fatty Acyls
D-erythro-C18-Sphingosine	Sphingosine	282.29	282.28	265; 121	0.7	Positive	Lipids	Organonitrogen compounds
Luteolin	LT	287.06	287.06		0.7	Positive	Flavones	Flavonoids
9-hydroxy-10E,12Z,15Z-octadecatrienoic acid	9-HOTrE	293.21	293.21	275; 171; 121	0.7	Positive	Fatty Acyls	Fatty Acyls
5-Hydroxy-6,7-dimethoxyflavone	Mosloflavone	299.06	299.09		0.7	Positive	Carboxylic acids and derivatives	Isoflavonoids
Quercetin	QUE	303.04	303.05		0.7	Positive	Carboxylic acids and derivatives	Flavonoids
4,4'-Methylenebis(2,6-diethylaniline)		311.25	311.25		0.7	Positive	Lipids	Organooxygen compounds
7-Hydroxycoumarin glucoside	Skimmin	325.10	325.09	163	0.7	Positive	Carboxylic acids and derivatives	Coumarins and derivatives
4-[(E)-3-(3,4-dihydroxyphenyl)prop-2-enoyl]oxy-2,3-dihydroxy-2-		330.12	330.12	181; 163; 145; 135; 117; 107; 89	0.7	Positive	Cinnamic acids and derivatives	Cinnamic acids and derivatives

methylbutanoic acid								
4',5,7-Trihydroxy-3,6-dimethoxyflavone	3,6-Dimethoxyapigenin	331.07	331.08	315	0.7	Positive	Phenols	Flavonoids
5,12-Dihydroxy-6,8,10,14-eicosatetraenoic acid	5,12-DiHETE	335.25	335.22	317	0.7	Positive	Lipids	Fatty Acyls
Chlorogenic acid	CGA	337.09	337.09	193; 181; 163; 145; 135; 117; 107; 89	0.7	Positive	Carboxylic acids and derivatives	Cinnamic acids and derivatives
Cyclohexanecarboxylic acid, 1,3,4-trihydroxy-5-[[(2E)-3-(4-hydroxyphenyl)-1-oxo-2-propen-1-yl]oxy]-, (1R,3R,4S,5R) (3-p-Coumaroylquinic acid)	3-CoQA	339.11	339.11	147; 119; 91	0.7	Positive	Carboxylic acids and derivatives	Cinnamic acids and derivatives
Monolinolenin (9c,12c,15c)		353.27	353.27	335; 279; 261; 243; 233; 205; 191; 187; 173; 163; 159; 149; 145; 133; 121; 109; 107; 95; 93	0.7	Positive	Lipids	Fatty Acyls
Caffeoylquinic acid	CQA	355.10	355.10	163; 145; 135; 117	0.7	Positive	Carboxylic acids and derivatives	Cinnamic acids and derivatives
(2R)-3-(3,4-dihydroxyphenyl)-2-[(E)-3-(3,4-dihydroxyphenyl)prop-2-enoyl]oxypropanoic acid (Rosmarinic acid)	RosA	361.14	361.10	145; 135; 117; 107; 89	0.7	Positive	Carboxylic acids and derivatives	Cinnamic acids and derivatives
Cyclohexanecarboxylic acid, 1,3,5-trihydroxy-4-[[(2E)-3-(4-hydroxy-3-methoxyphenyl)-1-oxo-2-propen-1-yl]oxy]-, (1alpha,3alpha,4alpha,5beta) (4-O-Feruloyl-D-quinic acid)	4-O-FQA	369.12	369.12	177; 149; 145; 117; 89	0.7	Positive	Carboxylic acids and derivatives	Cinnamic acids and derivatives
(1S,3R,4R,5R)-3-[(E)-3-(3,4-dihydroxyphenyl)prop-2-enoyl]oxy-1,4,5-trihydroxycyclohexane-1-carboxylic acid (Chlorogenic acid)		372.13	372.13	181; 163; 145; 135; 117; 89	0.7	Positive	Carboxylic acids and derivatives	Cinnamic acids and derivatives
beta-D-Glucopyranoside, 2-(1,3-benzodioxol-5-yl)-3-hydroxypropyl		381.07	381.12		0.7	Positive	Benzene and substituted	Organooxygen compounds

							derivatives	
Isovitexin	IVT	431.10	431.10	311	0.7	Positive	Lipids	Flavonoids
Isoorientin	ISOO	449.11	449.11	431; 413; 395; 385; 367; 329; 311; 299	0.7	Positive	Lipids	Flavonoids
Luteolin-6-C-glucoside (Isoorientin)	ISOO	449.12	449.11	431; 413; 395; 383; 353; 329; 299	0.7	Positive	Carboxylic acids and derivatives	Flavonoids
(3R,5R)-3,5-bis[[(E)-3-(3,4-dihydroxyphenyl)prop-2-enoyl]oxy]-1,4-dihydroxycyclohexane-1-carboxylic acid (3,5-Dicaffeoylquinic acid)	3,5-diCQA	499.12	499.12	181; 163; 145; 135; 117; 89	0.7	Positive	Carboxylic acids and derivatives	Cinnamic acids and derivatives
Flavone base + 4O, C-Hex-dHex		595.16	595.17	577; 449; 431; 413; 395; 367; 339; 329; 299	0.7	Positive	Flavonoid glycosides	Flavonoids
4-Hydroxy-6-Methylpyran-2-One		127.00	127.04	99; 87; 71	0.7	Negative	Carboxylic acids and derivatives	Pyrans
Indoxyl Sulfate		134.03	134.06	106	0.7	Negative	Imidazopyrimidi-nes	Organic sulfuric acids and derivatives
Phenylacetic acid		135.04	135.04	107; 93	0.7	Negative	Benzene and substituted derivatives	Benzene and substituted derivatives
(4S,5Z,6S)-4-(2-methoxy-2-oxoethyl)-5-[2-[(E)-3-phenylprop-2-enoyl]oxyethylidene]-6-[(2S,3R,4S,5S,6R)-3,4,5-trihydroxy-6-(hydroxymethyl)oxan-2-yl]oxy-4H-pyran-3-carboxylic acid		149.01	149.06	122	0.7	Negative	Phenols	Organooxygen compounds
Daphnetin	Daph	179.03	179.03	161; 135	0.7	Negative	Coumarins and derivatives	Coumarins and derivatives
D-(-)-Quinic acid	QA	191.05	191.06	85	0.7	Negative	Benzene and substituted derivatives	Organooxygen compounds
7-Methoxy-4-methylcoumarin	4-Methylherniarin	191.05	191.07	135	0.7	Negative	Benzene and	Coumarins and

							substituted derivatives	derivatives
trans-3-Hydroxycotinine	THOC	193.05	193.10	175; 149; 134	0.7	Negative	Carboxylic acids and derivatives	Pyridines and derivatives
6-Hydroxy-7,7-dimethyl-7,8-dihydroimidazo[1,2-a][1,3,5]triazine-2,4(3H,6H)-dione	Terbuthylazine-TP	197.12	197.07	169; 154; 127; 111; 85	0.7	Negative	Carboxylic acids and derivatives	Diazines
3-Isobutylhexahydropyrrolo[1,2-a]pyrazine-1,4-dione	Cyclo(L-Leu-L-Pro)	211.13	211.15	195	0.7	Negative	Amino acids, peptides and analogues	Carboxylic acids and derivatives
(7R,8R)-7,8-dihydroxy-3,5,7-trimethyl-8H-isochromen-6-one		245.04	245.08	227; 185; 153	0.7	Negative	Benzene and substituted derivatives	Organonitrogen compounds
Chrysin		253.09	253.05		0.7	Negative	Flavonoids	Flavonoids
Linolenic acid	LA	277.22	277.24	233	0.7	Negative	Fatty Acyls	Fatty Acyls
5-(2-Methyl-3-(pyridin-3-yl)-1H-indol-1-yl)pentanoic acid		309.20	309.16	291; 273; 263; 247; 223	0.7	Negative	Indoles and derivatives	Benzene and substituted derivatives
Phenylbutazone	PBZ	309.20	309.16	211	0.7	Negative	Organooxygen compounds	Benzene and substituted derivatives
Hydroquinidine	HQ	327.21	327.21	307	0.7	Negative	Arylsulfates	Alkaloids
4',5,7-trihydroxy-3,6-dimethoxyflavone	3,6-Dimethoxyapigenin	329.07	329.07	314; 299; 271	0.7	Negative	Carboxylic acids and derivatives	Flavonoids
Chlorogenoquinone		351.07	351.07	191; 179; 161; 135	0.7	Negative	Benzene and substituted derivatives	Organooxygen compounds
1,7-bis(4-hydroxyphenyl)-5-methoxyheptan-3-one	Platyphylline	351.20	351.16	289; 107	0.7	Negative	Flavonoids	Organooxygen compounds
Caffeoylquinic acid	CQA	353.08	353.09	191; 179; 173; 161; 135	0.7	Negative	Flavonoids	Cinnamic acids and derivatives
Chlorogenic Acid	CGA	354.28	354.31	191; 179; 173	0.7	Negative	Flavonoids	Cinnamic acids and derivatives
Feruloylquinic acid	FQA	367.10	367.10	193; 173; 149; 134	0.7	Negative	Steroids and	Cinnamic acids and derivatives

							steroid derivatives	
(1S,4aS,7aS)-1-(beta-D-Glucopyranosyloxy)-7-(hydroxymethyl)-1,4a,5,7a-tetrahydrocyclopenta[c]pyran-4-carboxylic acid	Geniposidic acid	397.13	397.11	235	0.7	Negative	Carboxylic acids and derivatives	Organooxygen compounds
2H-Naphtho[2,1-c]pyran-7-carboxylic acid, 2-(3-furanyl)-1,4,4a,5,6,6a,7,10,10a,10b-decahydro-7-hydroxy-6a,10b-dimethyl-4-oxo-, methyl ester, (2S,4aS,6aR,7R,10aR,10bR)		397.13	397.16		0.7	Negative	Carboxylic acids and derivatives	Organic compounds
Hexose + C13H17O3	Corchoionoside B	399.16	399.17	219; 175; 160	0.7	Negative	Organooxygen compounds	Organooxygen compounds
Methyl 3,5-dimethoxy-2-[5-methoxy-4-oxo-6-[(E)-prop-1-enyl]pyran-3-carbonyl]benzoate	Methylfunicone	411.07	411.11		0.7	Negative	Flavonoids	Pyrans
Naftopidil	NAF	415.16	415.20		0.7	Negative	Benzoxepines	Diazinanes
beta-D-Glucopyranoside, 2-phenylethyl 2-O-beta-D-xylopyranosyl-		439.11	439.16	307	0.7	Negative	Carboxylic acids and derivatives	Organooxygen compounds
11,15-Diene-14-carboxylic acid, 3,15,19-trihydroxy-4,4,8,12,16-pentamethyl-17-oxo-, methyl ester		445.23	445.26		0.7	Negative	Organooxygen compounds	Glycerolipids
Luteolin-6-C-glucoside		447.08	447.09	429; 357; 327	0.7	Negative	Carboxylic acids and derivatives	Flavonoids
Quercetin-3-O-galactoside	Hyperoside	463.09	463.09	301	0.7	Negative	Flavonoids	Flavonoids
Emetine		481.27	481.31		0.7	Negative	Lipids	Alkaloids
PG(16:1/0:0)		482.27	482.26		0.7	Negative	Lipids	Glycerophospholipids
MGMG 18:3		513.31	513.30	513; 277; 253; 235	0.7	Negative	Organooxygen compounds	Glycerolipids
beta-D-Glucopyranoside, 2-methoxy-4-[tetrahydro-3a,6a-dihydroxy-4-(4-hydroxy-3-methoxyphenyl)-1H,3H-		575.14	575.17		0.7	Negative	Lipids	Furanoid lignans

furo[3,4-c]furan-1-yl]phenyl								
2-(3,4-dihydroxyphenyl)-5-hydroxy-3,7-bis[[(2S,3R,4R,5R,6S)-3,4,5-trihydroxy-6-methyloxan-2-yl]oxy]chromen-4-one	Quercetin 3,7-dirhamnoside	593.16	593.15	447	0.7	Negative	Flavonoids	Flavonoids
DGMG 18:3		721.35	721.36	675; 415; 397; 287; 112	0.7	Negative	Organooxygen compounds	Glycerolipids
Smiglaside C + 1Acetyl		861.25	861.25		0.7	Negative	Hexacarboxylic acids and derivatives	Glycerolipids

Table S2. Metabolites manually annotated based on searching the calculated molecular formulas against the DNP, Chemspider, SorgCyc, PlantCyc and KEGG databases, confirming the metabolite structure by inspecting the fragmentation pattern, and comparison with annotated metabolites in published literature, particularly in (Kang et al., 2016).

Metabolite Annotation	Metabolite Abbreviation	RT (min.)	Precursor mass (*m/z*)	Diagnostic fragments (*m/z*)	Ionisation Mode	ClassyFire Class Annotation	Manually assigned Chemical Class Terms
Galloyl-hexose	Glucogallin	1.68	331.063	168; 125	Negative	Benzene and substituted derivatives	Tannins
Isocitric acid		1.85	191.017	173; 129; 111; 85	Negative	Benzene and substituted derivatives	Carboxylic acids and derivatives
Caffeoyl-glucaric acid	Caffeoylglucarate	1.85	371.057	209; 191; 179	Negative	Benzene and substituted derivatives	Carboxylic acids and derivatives
Hydroxycitric acid	HCA	2.20	207.012	191; 189; 127	Negative	Benzene and substituted derivatives	Carboxylic acids and derivatives
DIBOA-glucoside		2.29	342.081	180; 153	Negative	Benzene and substituted derivatives	Organooxygen compounds
5-Coumaroylquinic acid	5-CoQA	3.36	337.086	191; 163	Negative	Organooxygen compounds	Cinnamic acids and derivatives
Glucosyl-apigenin	Apigenin 7-O-beta-D-glucoside	3.53	431.153	269	Negative	Flavonoids	Flavonoids
5-Caffeoylquinic acid	5-CQA	3.65	353.084	191	Negative	Flavonoids	Cinnamic acids and derivatives
Sinapoyl-D-glucose		4.18	385.109	223; 205; 179	Negative	Organooxygen compounds	Cinnamic acids and derivatives
Clovamide		4.43	358.091	222; 178	Negative	Flavonoids	Carboxylic acids and derivatives
1,3-Dicaffeoylquinic acid	1,3-diCQA	4.49	515.118	353; 191; 179	Negative	Flavonoids	Cinnamic acids and derivatives
2-Feruloylhydroxycitric acid		4.8	383.059	189; 127; 33; 83	Negative	Flavonoids	Carboxylic acids and derivatives
3-Feruloylquinic acid	3-FQA	4.83	367.099	193; 173; 134	Negative	Steroids and steroid derivatives	Cinnamic acids and derivatives
Isoschaftoside		4.92	563.138	473; 443; 413; 383; 353	Negative	Carboxylic acids and derivatives	Flavonoids

Neohesperidin	NEO	5.23	609.146	301; 300; 293	Negative	Benzene and substituted derivatives	Flavonoids
Rutin (Quercetin-*O*-hexoside-O-rhamnoside)	RUT	5.54	609.146	301; 300; 271; 255	Negative	Flavonoids	Flavonoids
Tricin-*O*-(5/7/4`)hexoside-*O*-(5/7/4`)rhamnoside		5.94	637.247	439; 399; 329	Negative	Organooxygen compounds	Flavonoids
Kaempferol-7-*O*-hexoside		6.27	447.088	285; 284; 255	Negative	Flavonoids	Flavonoids
Apigenin-*C*-hexoside-*C*-rhamnoside		6.33	577.152	473; 437; 413; 357; 327; 298	Negative	Flavonoids	Flavonoids
Maysin	Mys	6.74	575.139	473; 411; 337; 298; 285	Negative	Lipids	Flavonoids
Diosmetin-7-O-rutinoside		7.33	607.163	299; 284	Negative	Lipids	Flavonoids
Dihydroxy-dodecadienoic acid		9.34	227.127	183; 165; 160	Negative	Organooxygen compounds	Fatty Acyls
Alpha-D-glucopyranosyl-nonadecandiol		13.24	577.266	299; 225	Negative	Lipids	Glycerolipids
Palmitoylglycerol-phosphate		14.33	431.216	295; 277; 195; 152	Negative	Lipids	Glycerophospholipids
Isoleucine	Ile	1.37	132.107	86	Positive	Carboxylic acids and derivatives	Amino acids and derivatives
Phenylalanine	Phe	1.90	166.089	120; 103	Positive	Carboxylic acids and derivatives	Amino acids and derivatives
Tryptophan	Trp	2.84	205.100	188	Positive	Carboxylic acids and derivatives	Amino acids and derivatives
7-Hydroxycoumarin	Umbelliferone	3.23	163.045		Positive	Coumarins and derivatives	Coumarins and derivatives
Coumarin		4.02	147.044	91	Positive	Coumarins and derivatives	Coumarins and derivatives
Ascorbic acid	ASC	4.32	177.057	145	Positive	Carboxylic acids and derivatives	Dihydrofurans
DIMBOA		4.43	212.060	193; 177; 166	Positive	Benzene and substituted derivatives	Organooxygen compounds
Dimethoxycinnamic acid	Dimethylcaffeic acid	5.26	209.158	177; 163; 149	Positive	Carboxylic acids and derivatives	Cinnamic acids and derivatives
Kaempferol-3-rutinoside		7.11	594.188	577; 449; 432;	Positive	Flavonoids	Flavonoids

				413; 287			

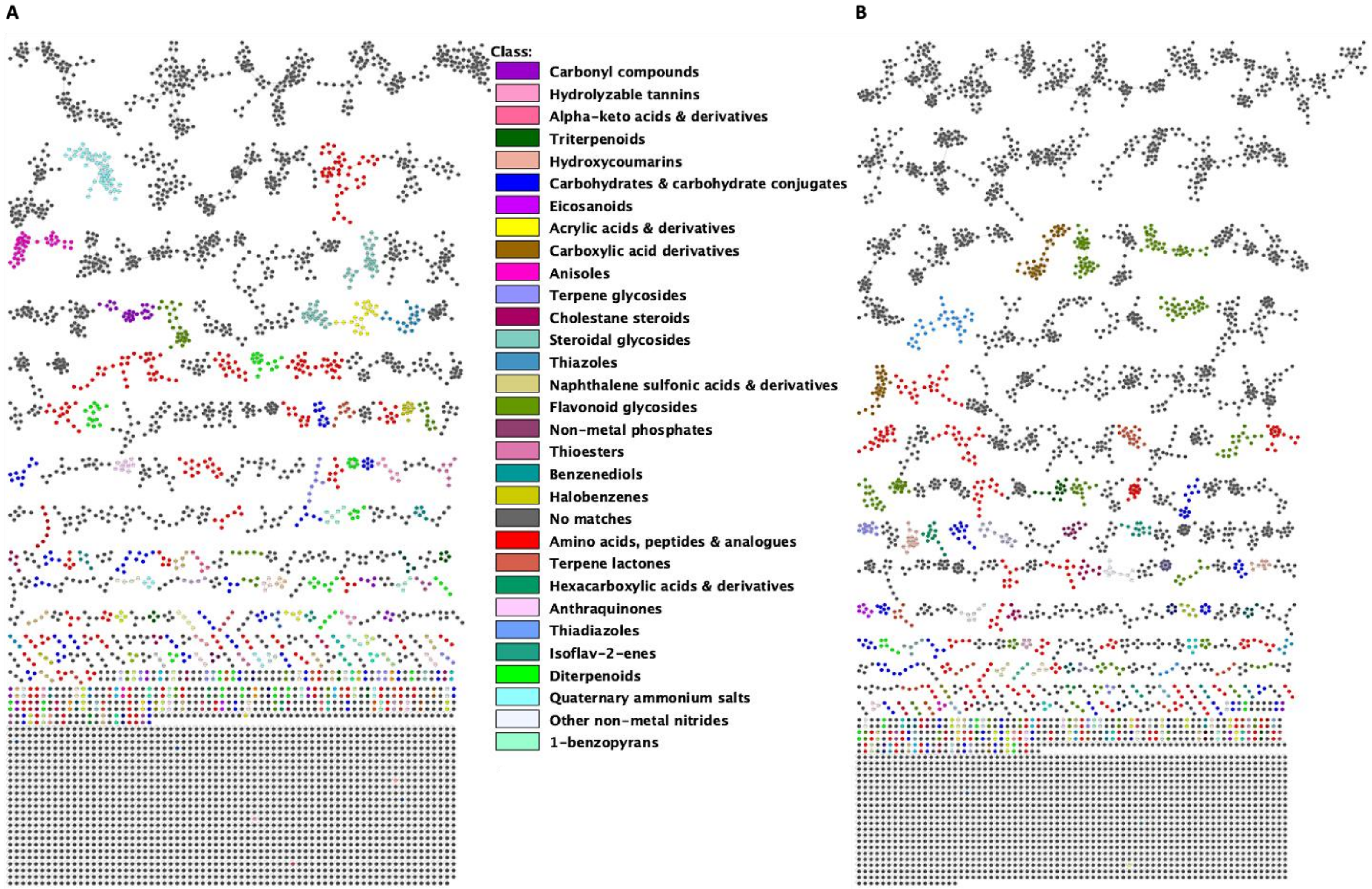

Figure S3. MolNetEnhancer enriched feature-based molecular networks of (**A**) positive electrospray ionisation (ESI$^+$) and (**B**) negative electrospray ionisation (ESI$^-$) MS/MS spectra obtained from maize leave extracts. The MolNetEnhancer enriched molecular networks depict structurally similar nodes as molecular families/clusters. GNPS spectral library matched annotations and *in silico* NAP annotations were used to predict chemical compound subclass annotations, represented by coloured nodes (ClassyFire subclass ontology terms) with nodes without subclass annotation coloured grey.

Table S3. Discriminating features (*m/z* ions) selected using OPLS-DA modelling. The features were selected from OPLS-DA loading S-plots, and based on descriptive statistics, these features have a *p*-value < 0.05, the fold changes describe the differential changes between treatment groups. These include (**i**) normal well-watered conditions, T1 *vs* T3 = control group *vs* soil applied biostimulant; T1 *vs* T5 = control *vs* foliar applied biostimulant; (**ii**) under drought stress conditions, T2 *vs* T4 = control *vs* soil applied biostimulant; and T2 *vs* T6 = control *vs* foliar applied biostimulant.

	Metabolite Annotation	Precursor mass (m/z)	Ionisation/ESI mode	Fold change			
				T1 *vs* T3	T1 *vs* T5	T2 *vs* T4	T2 *vs* T6
1	Chlorogenic acid	372.13	Positive	10.4944	13.2289	--	2.50993
2	Caffeic acid	181.06	Positive	12.129	11.8128	12.8248	--
3	3-p-Coumaroylquinic acid	339.11	Positive	6.53507	4.84211	--	
4	4-O-Feruloyl-D-quinic acid	369.12	Positive	3.18574	3.20065	--	1.87538
5	Isoorientin	449.11	Positive		2.97742	--	2.10284
6	4-[(E)-3-(3,4-dihydroxyphenyl)prop-2-enoyl]oxy-2,3-dihydroxy-2-methylbutanoic acid	330.12	Positive	3.14916	2.78827	--	--
7	Isoleucine	132.107	Positive	3.61817	2.72072	2.21818	2.04309
8	3-Nitro-L-tyrosine	225.04	Positive	0.7611	2.5475	2.23178	4.04064
9	4-Coumaric acid	165.06	Positive	2.24436	2.42359	--	1.87526
10	DIMBOA	212.060	Positive	3.0041	2.19735	--	--
11	Rosmarinic acid	361.14	Positive		2.13027	2.04633	2.06454
12	Isoferulic acid	195.07	Positive	1.58899	1.91499	1.68017	1.7849
13	Oleamide	282.28	Positive	1.27436	1.845	--	0.65125
14	Caffeoylquinic acid	355.10	Positive	1.48372	1.8049	--	1.38004
15	3,5-Dicaffeoylquinic acid	499.12	Positive	0.792413	1.61802	1.65862	--
16	Flavone base + 4O, C-Hex-dHex	595.16	Positive	--	1.54967	1.5001	--
17	Tryptophan	205.100	Positive	1.54858	1.5408	1.81519	1.51303
18	Rosmarinic acid	163.04	Positive	1.44044	1.46159	--	--
19	Chlorogenic acid	337.09	Positive	0.71981	1.37207	1.39695	--
20	Phenylalanine	166.089	Positive	1.62253	1.23116	--	--
21	Coumarin	147.044	Positive	1.25865	1.15174	0.697786	0.801883
22	Ascorbic acid	177.057	Positive	1.19515	1.11454	---	0.829177
23	Monolinolenin	353.27	Positive	1.11524	1.09418	1.49741	1.48348
24	1-Naphthol	145.03	Positive	0.840155	0.850788	0.808124	0.750667
25	Pinolenic acid	261.22	Positive	--	0.850328	1.46484	1.3202
26	2-Naphthol	145.03	Positive	--	0.828374	0.818342	0.843147
27	(4S,5Z,6S)-4-(2-methoxy-2-oxoethyl)-5-[2-[(E)-3-phenylprop-2-enoyl]oxyethylidene]-	149.03	Positive	--	0.82761	0.67943	0.634595

	6-[(2S,3R,4S,5S,6R)-3,4,5-trihydroxy-6-(hydroxymethyl)oxan-2-yl]oxy-4H-pyran-3-carboxylic acid						
28	beta-D-Glucopyranoside, 2-(1,3-benzodioxol-5-yl)-3-hydroxypropyl	381.07	Positive	--	0.753659	--	--
29	9-HOTrE	293.21	Positive		0.744466	--	2.14705
30	3,6-Dimethoxyapigenin	331.07	Positive	0.628963	0.740743	0.740934	0.764076
31	Isovitexin	431.10	Positive	--	0.688611	--	0.592188
32	4,4'-Methylenebis(2,6-diethylaniline)	311.25	Positive	0.710754	0.647564	--	--
33	Adenine	136.06	Positive	0.729843	0.574953	0.673037	0.594337
34	Mosloflavone	299.06	Positive		0.361895	0.509342	0.630973
35	Phenethylamine	122.10	Positive	0.199693	0.130627	--	--
36	Unknown	609.2	Positive	39.3651	115.624	--	0.251585
37	Unknown	663.1975	Positive	28.409	93.8611	5.05567	14.612
38	Unknown	361.2	Positive	---	42.9979	--	7.22383
39	Unknown	372.2	Positive	---	42.0263	--	--
40	Unknown	597.2379	Positive	13.375	30.0117	4.25424	17.9952
41	Unknown	480.1792	Positive	--	19.5221	--	--
42	Unknown	333.2	Positive	9.2414	14.6326	---	--
43	Unknown	529.2566	Positive		130.471	---	7.12296
44	Unknown	701.4	Positive	11.8758	13.5887	6.05054	11.617
45	Unknown	595.2	Positive		13.5452	15.0596	44.0796
46	Unknown	469.2	Positive	11.4584	12.8629	8.44296	5.64191
47	Unknown	969.3854	Positive	5.57716	12.3445	--	0.505624
48	Unknown	659.2221	Positive	3.41814	12.291	3.70946	13.4256
49	Unknown	683.1	Positive	6.78151	11.5184	2.60385	--
50	Unknown	621.2	Positive	14.2503	11.133	--	2.05054
51	Unknown	669.2	Positive	4.70576	10.7753		--
52	Unknown	489.2	Positive	181.667	10.467	--	---
53	Unknown	558.099	Positive	0.131532	8.53154	---	---
54	Unknown	458.3238	Positive	--	8.46583	2.27218	3.68438
55	Unknown	373.2232	Positive	8.81935	8.23571	--	---
56	Unknown	275.2063	Positive	--	8.1932	--	3.12261
57	Unknown	401.1581	Positive	4,19974	7.81104	--	--
58	Unknown	316.2179	Positive	7.06704	7.48433	4.87977	2.17392
59	Unknown	355.1523	Positive	17.7554	7.47921	--	---
60	Unknown	387.1369	Positive	--	6.21781	---	6.72439

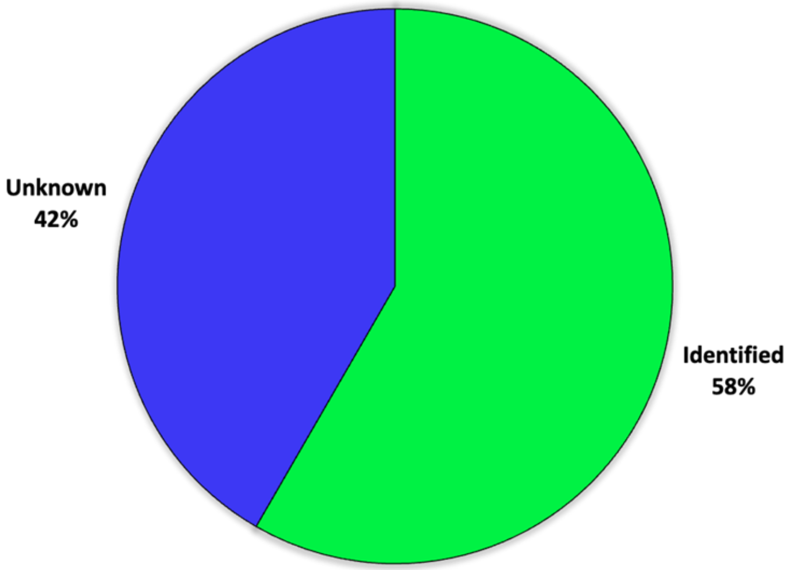

Figure S4. A pie chart of selected differential metabolite features, depicting the percentage of annotated metabolites using the MN approach.

Table S4. Significantly altered metabolic pathways in seaweed-based biostimulant treated maize plants under normal and drought stress conditions, with their metabolite hits/matches, calculated p-value (Raw p), Holm-Bonferroni method adjusted p-value (Holm adjust) and pathway topology analysis calculated impact value.

Pathway	Hits	Raw p	Holm adjust	Impact
Phenylalanine metabolism	2	8.0606e-10	1.5315e-08	0.61539
Stilbenoid, diarylheptanoid and gingerol biosynthesis	2	0.0083225	0.049935	0.25
alpha-Linolenic acid metabolism	1	0.080037	0.40019	0.11368
Phenylpropanoid biosynthesis	7	3.8338e-05	0.00057506	0.17468
Glyoxylate and dicarboxylate metabolism	1	0.58756	1	0.10393
Arginine and proline metabolism	1	0.0018983	0.017085	0.06649
Citrate cycle (TCA cycle)	1	0.58756	1	0.04508
Flavonoid biosynthesis	4	0.0069413	0.048589	0.05138
Phenylalanine, tyrosine and tryptophan biosynthesis	1	1.9623e-11	4.1209e-10	0.0015
Ubiquinone and other terpenoid-quinone biosynthesis	1	0.00033935	0.0040721	0.00102
Tropane, piperidine and pyridine alkaloid biosynthesis	1	1.9623e-11	4.1209e-10	0
Purine metabolism	1	2.6986e-08	4.8574e-07	0
Zeatin biosynthesis	1	2.6986e-08	4.8574e-07	0
Aminoacyl-tRNA biosynthesis	2	9.3596e-07	1.4975e-05	0
Valine, leucine and isoleucine degradation	1	5.8524e-05	0.00081933	0
Valine, leucine and isoleucine biosynthesis	1	5.8524e-05	0.00081933	0
Ascorbate and aldarate metabolism	1	0.00062287	0.0062287	0
Arachidonic acid metabolism	1	0.0043129	0.034503	0
Biosynthesis of unsaturated fatty acids	1	0.080037	0.40019	0
Flavone and flavonol biosynthesis	4	0.17864	0.53592	0

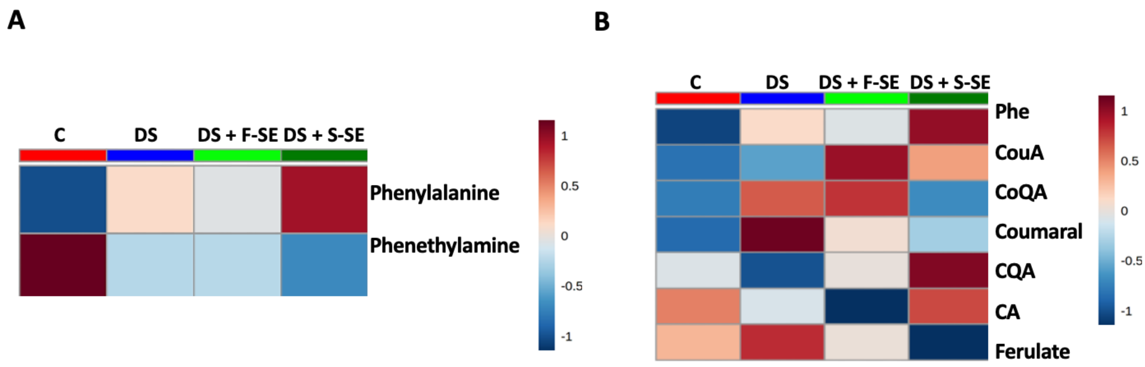

Figure S5. Heatmaps displaying differential qualitative alterations in the concentrations of some of the (A) phenylalanine metabolism and (B) phenylpropanoid biosynthesis pathway metabolites **Abbreviation:** C, Control (no stress; no seaweed extract biostimulant); DS, Drought stress (stress; no seaweed extract biostimulant); DS + F-SE, Drought stress with foliar applied seaweed extract biostimulant; DS + S-SE, Drought stress with soil applied seaweed extract biostimulant; Phe, Phenylalanine; CouA, Coumaric acid; CoQa, Coumaroylquinic acid; Coumaral, Coumaraldehyde; CQA, Caffeoylquinic acid; CA, Caffeic acid.

Table S5. Height (cm) of maize plants with and without seaweed-based biostimulant treatment under normal and drought stress conditions, with the calculated p-value, 95% probability level (Fisher's LSD test) and cross-validation percentage (CV%). Measurements indicate the effect of the seaweed-based biostimulant treatment on the maize plant's height under normal and drought stress conditions.

**Treatment Description**	**1 Day after treatment**	**3 Days after treatment**	**7 Days after treatment**	**14 Days after treatment**
Control (No seaweed extract, no stress)	13.66	14.54	15.80 a	21.34 c
Control (No seaweed extract with stress)	13.70	14.28	16.46 ab	16.83 a
Soil applied seaweed extract with no stress	14.56	14.58	17.74 b	23.02 d
Soil applied seaweed extract with stress	14.36	14.30	15.94 a	16.82 a
Foliar applied seaweed extract with no stress	14.26	14.82	15.86 a	21.79 cd
Foliar applied seaweed extract with stress	14.58	14.16	15.74 a	18.15 b
**Descriptive statistics**				
Total n	30	30	30	30
Mean	14.19	14.45	16.26	19.66
Std Dev	0.9209	1.370	1.285	2.669
p-value	0.8205	0.9441	0.0760	0.0192
LSD (α=0.05)	1.203	1.938	1.530	1.239
CV%	6.491	9.480	7.902	13.58

Means within a column with the **same letter(s)** are **not significantly** different at the 95% probability level (Fisher's LSD test)

Table S6. Diameter of maize plants with and without seaweed-based biostimulant treatment under normal and drought stress conditions, with the calculated p-value and 95% probability level (Fisher's LSD test). Measurements indicate the effect of the seaweed-based biostimulant treatment on the maize plant's diameter under normal and drought stress conditions.

**Treatment Description**	**1 Day after treatment**	**3 Days after treatment**	**7 Days after treatment**	**14 Days after treatment**
Control (No seaweed extract, no stress)	11.65 ab	12.34	12.17 ab	13.67 ab
Control (No seaweed extract with stress)	11.33 a	12.44	12.61 ab	12.78 a
Soil applied seaweed extract with no stress	12.34 b	11.93	13.07 b	14.26 b
Soil applied seaweed extract with stress	11.92 ab	11.83	12.34 ab	12.82 ab
Foliar applied seaweed extract with no stress	12.27 ab	11.90	12.49 ab	13.74 ab
Foliar applied seaweed extract with stress	11.39 a	12.11	12.05 a	13.70 ab
**Descriptive statistics**				
Total n	30	30	30	30
Mean	11.82	12.09	12.46	13.50
Std Dev	0.773	0.806	0.761	1.160
p-value	0.6608	0.9175	0.2134	0.3943
LSD (α=0.05)	0.946	1.108	0.978	1.474
CV%	6.537	6.668	6.111	8.595

Means within a column with the **same letter(s)** are **not significantly** different at the 95% probability level (Fisher's LSD test)

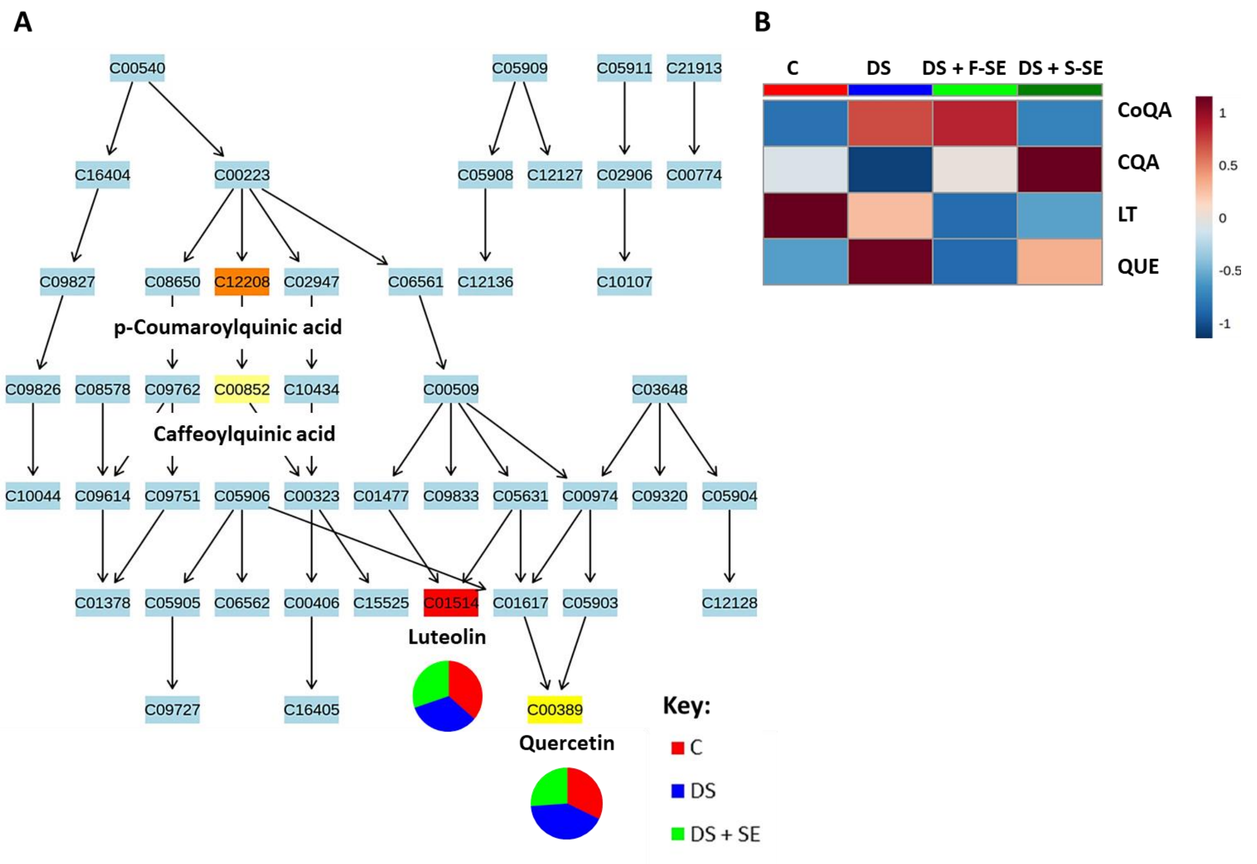

Figure S6. Summary of Metabolomics Pathway Analysis (MetPA) outputs. **(A)** Topological characteristics of flavonoid biosynthesis pathway including quantification levels of Luteolin and Quercetin. **(B)** Heatmaps displaying differential qualitative alterations in the concentrations of some of the flavonoid biosynthesis pathway metabolites **Abbreviation:** C, Control (no stress; no seaweed extract biostimulant); DS, Drought stress (stress; no seaweed extract biostimulant); DS + SE, Drought stress with seaweed extract biostimulant; CoQa, Coumaroylquinic acid; Coumaral, Coumaraldehyde; CQA, Caffeoylquinic acid; LT, Luteolin and QUE, Quercertin.

Table S7. Seaweed extract biostimulant application rates (L/ha) applied to the plant sample groups.

Treatment	Treatment Description	Application Rate (L/ha)
1	Control (No seaweed extract, no stress)	0
2	Control (No seaweed extract with stress)	0
3	Soil applied seaweed extract with no stress	1
4	Soil applied seaweed extract with stress	1
5	Foliar applied seaweed extract with no stress	1
6	Foliar applied seaweed extract with stress	1
